# Supramolecular Materials as Solid-Phase Microextraction Coatings in Environmental Analysis

**DOI:** 10.3390/molecules29122802

**Published:** 2024-06-12

**Authors:** Nicolò Riboni, Erika Ribezzi, Federica Bianchi, Maria Careri

**Affiliations:** Department of Chemistry, Life Sciences and Environmental Sustainability, University of Parma, Parco Area Delle Scienze 17/A, 43124 Parma, Italy; erika.ribezzi@unipr.it (E.R.); careri@unipr.it (M.C.)

**Keywords:** solid-phase microextraction, metal organic frameworks, covalent organic frameworks, supramolecular receptors, cyclodestrins, environmental monitoring

## Abstract

Solid-phase microextraction (SPME) has been widely proposed for the extraction, clean-up, and preconcentration of analytes of environmental concern. Enrichment capabilities, preconcentration efficiency, sample throughput, and selectivity in extracting target compounds greatly depend on the materials used as SPME coatings. Supramolecular materials have emerged as promising porous coatings to be used for the extraction of target compounds due to their unique selectivity, three-dimensional framework, flexible design, and possibility to promote the interaction between the analytes and the coating by means of multiple oriented functional groups. The present review will cover the state of the art of the last 5 years related to SPME coatings based on metal organic frameworks (MOFs), covalent organic frameworks (COFs), and supramolecular macrocycles used for environmental applications.

## 1. Introduction

Since its development in 1989 by Arthur and Pawliszyn [1], solid-phase microextraction (SPME) has attracted increasing attention, being nowadays considered one of the most widely applied sample pretreatment techniques [2,3,4]. Its unique features, integrating sampling, extraction, preconcentration, and desorption into one single step, have provided noticeable advantages in terms of extraction efficiency, reduced sample handling, time consumption, and ease of use [5], thus enabling the development of fast, efficient, and high-throughput analytical methods [6,7]. Both the principles and the different geometries of SPME technologies and devices have been discussed in already-published reviews [2,3,8]. Environmental friendliness is another key feature of SPME, being that the reduced consumption of organic solvents, the prevention of waste generation, the automation, miniaturization, and possibility of in situ measurements are some of the criteria that perfectly comply with the principles of green analytical chemistry [9,10]. Therefore, SPME has been widely applied in different research fields, including environmental monitoring [6,11,12], food [13,14], forensics [15,16], pharmaceutical [15,17], and bio-analysis [4,15,18,19,20]. 

The performance of SPME in terms of selectivity and extraction efficiency is mainly related to the absorption/adsorption capacity of the coating [5,21]. Commercial coatings such as polydimethylsiloxane (PDMS), polydivinylbenzene (DVB), polyacrylate (PA), carboxen (CAR), and their combinations are usually characterized by low selectivity and extraction efficiency. To overcome these limitations, molecular hosts as well as nanostructured and porous materials able to promote an enhanced selectivity via size-exclusion mechanisms and oriented intramolecular interactions [6,21,22] have been proposed as novel SPME coatings, providing more selective and efficient solutions [5,7,23,24,25]. In this context, the functionalization of the material can both strengthen the interactions between the materials and the target compounds or improve analyte segregation.

Owing to the designed 3D architecture and adsorption capacity driven by chemical and size synergistic complementarity, supramolecular receptors have emerged as alternative coatings cable of strongly affecting both the selectivity and extraction capabilities of SPME. The aim of the present review is to cover the state of the art of the last 5 years, summarizing the main advances in the development of SPME coatings based on metal organic frameworks (MOFs), covalent organic frameworks (COFs), and supramolecular macrocycles, including cyclodextrins (CDs), calixarenes, and cavitands, for environmental monitoring. In vivo SPME applications are not discussed since reviews focused on the matter have been very recently published [26,27,28].

## 2. Supramolecular-Based Coatings: Deposition Methods

**Figure 1:**
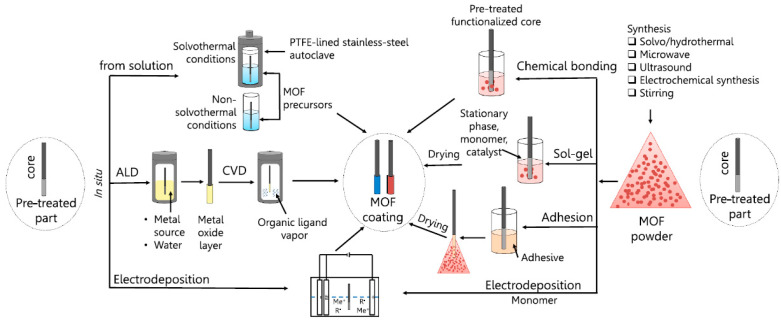
Schematic representation of the different approaches used for the development of supramolecular-based SPME coatings. Reprinted with permission from Omarova et al. [29].

Different approaches have emerged to develop SPME coatings using supramolecular materials, depending on both the nature of the adsorbent material and on the sampling conditions. It is known that the proper planning of the synthetic strategy plays a key role in guaranteeing the availability of the active binding sites together with the thermal, mechanical, and chemical stability of the coating [7,29,30,31]. The first step to be performed is the activation of the supporting material: to this aim, different treatments have been proposed, including ultrasonic treatment using organic solvents, etching using mineral acids (HF, HCl, HNO_3_), or deposition of a sol–gel coating bearing amino moieties, e.g., (3-aminopropyl) triethoxysilane (APTES). Both the ultrasonic treatment and acidic etching are useful to clean the support of the fiber, remove passive layers, and expose the metal ions/active groups on the support surface to strengthen the adhesion between the coating and the substrate. According to Omarova et al. [29], the coating approaches can be divided in two main groups: (i) in situ deposition during framework development, allowing the synthesis of the material directly on the surface of the fiber; (ii) two-stage deposition, including the synthesis of the adsorbent material and the post-synthetic deposition (Figure 1). 

In situ growth (or deposition from solution), chemical vapor deposition (CVD), and atomic layer deposition (ALD) are the most commonly applied methodologies of in situ deposition. The main advantages of these approaches rely on the development of coatings containing only the supramolecular frameworks, thus increasing the extraction selectivity.

In situ growth is based on the immersion of the pretreated fiber in the reacting solution, allowing for the synthesis of the material directly onto its surface [7,29,30,31,32]. In this context, stainless-steel (SS) supports are preferred to fused silica and quartz substrates, being that these fibers very fragile. Compared to the other in situ approaches, this technique is simpler and does not require the use of expensive equipment. However, it is time-consuming and several parameters, including reaction time, concentration of reagents, and agitation speed, need to be optimized to obtain stable and reproducible coatings [29,30].

**Figure 2:**
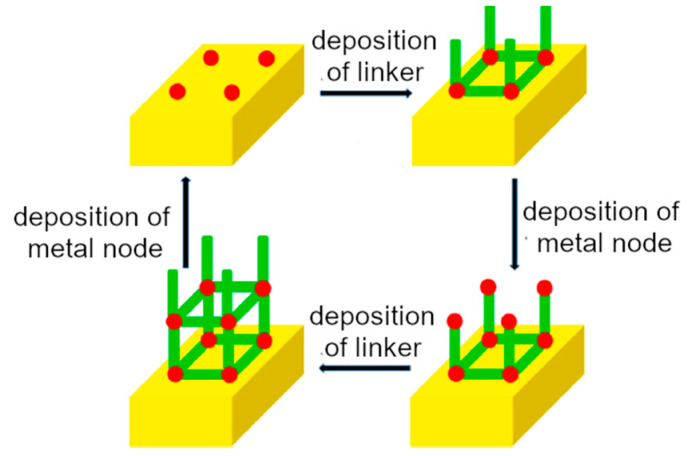
Schematic representation of the ALD process, edited from Zhou et al. [34].

CVD involves the deposition of the coating onto a heated substrate surface via the chemical reactions of evaporated precursors [33]. The structure, morphology, dimensions, and orientation of the coating can be finely tuned by changing the deposition conditions. This process is highly reproducible and can be performed at higher rates compared to the solution deposition [29]. Atomic layer deposition (ALD, or ALD–molecular layer deposition) is a nanotechnological modification of CVD, involving a vapor-phase layer-by-layer deposition of the coating materials onto a solid support exploiting gas–surface reactions [34]. The deposition process is divided into sequential reaction stages between the precursors and substrate, leading to the consecutive deposition of reactants (Figure 2): (i) in the first step a precursor (metallic center, polymeric material, or organic linker) is pulsed into a vacuum-heated chamber containing the fiber; (ii) after the deposition of the precursor layer, the unfunctionalized reactants are removed from the chamber; and (iii) the other precursors are purged onto the already-formed layer, leading to a formation of a second layer of the coating. The process is repeated until the desired coating is obtained. ALD provides a unique control on thin film formation in terms of conformality and uniformity, with atomic-level accuracy [29,34].

As for dual-stage deposition, different approaches have been proposed, including physical adhesion, the sol–gel technique, and chemical cross-linking [7,29,30,32].

The physical adhesion of a pre-synthesized material is the most commonly applied procedure [7,29,30,31,32]. Besides fiber pretreatment, the coating process consist of several steps: (i) the inert surface is immersed in an adhesive material (e.g., silicone-based sealants or epoxy glues) or pre-coated with a polymeric layer (usually polyacrylonitrile—PAN); (ii) the fiber is dipped in the fine powdered supramolecular material to obtain a coating with the intended thickness (multiple dipping cycles can be required); (iii) an additional layer of adhesive material can be applied to increase the mechanical stability of the fiber; and (iv) the fiber is dried and thermally conditioned to promote coating hardening, curing, and allowing for the evaporation of both the residual solvents and organic sub-products. Steps i–iii can be merged when the pretreated fiber is directly immersed into a solution containing the suspended material without the need of an adhesive medium [29,30,31]. In this case, the immersion–heating stages are usually performed multiple times to obtain the intended coating thickness. In physical adhesion, the adhesive (or the suspending solvent) used for developing the coating has a major impact on the SPME performance, affecting the thermal and mechanical stability of the material, as well as the resistance to organic solvents. The main advantage of this approach relies on its simplicity, cost-effectiveness, and versatility. Since the synthesis of the material is not affected by the deposition, microwave- or ultrasound-assisted reactions can be applied to obtain the supramolecular material [30]. The main drawback of this approach is related to the presence of adhesives or polymeric substances that could clog the pores or change the surface chemistry of the adsorbent, thus reducing the surface area or affecting the extraction efficiency and selectivity.

Concerning the sol–gel technique, it involves the embedding of the supramolecular materials into a gel network for coating deposition [29,30,35,36]. The pre-synthesized supramolecular material can be either dispersed in a sol–gel suspension or deposited after the gel formation onto the SPME support. The produced coatings are characterized by high homogeneity and uniformity as well as enhanced thermal, mechanical, and chemical resistance compared to the use of adhesives. In addition, the sol–gel process usually occurs under very mild conditions, and the thickness, composition, and morphology of the coating can be finely tuned by changing the concentration of the precursors, the dipping rate, the number of dipping cycles, and the drying temperature. The main drawback of this approach relies on the encapsulation of the supramolecular framework within the gel network, which can lead to the pores clogging and possible interactions between the silica gel and the compounds present in the sample.

Chemical cross-linking is based on the chemical bonding of the pre-synthesized supramolecular material with the fiber surface [25,29,30]. The fiber is pre-treated to obtain carboxylic or amino groups at the surface to be used as binding sites for the immobilization of the material. In this context, the frameworks are linked to the fiber with strong covalent bonds, thus providing high thermal and mechanical stability. Since no additive is required, selectivity is improved, and the extraction/desorption kinetics are higher than those achieved by the coatings obtained using other dual-stage deposition processes. However, chemical cross-linking could be a complex procedure and the amount of loaded material and the coating thickness could present a serious challenge to be tuned during coating deposition.

Finally, supramolecular coatings onto SPME fibers have been also obtained by using the electrochemistry approach: [29,31,37,38] in this case, both in situ and two-stage electrodeposition have been applied, depending on the synthetic approach, i.e., electrochemical reduction/oxidation or deposition of pre-synthesized supramolecular sorbents or by using conductive polymers such as polyaniline (PANI), polypyrrole (PPy), or polythiophene. The main advantages of this process rely on its high efficiency, repeatability, and ease of control, thus allowing the acquisition of coatings characterized by uniform thicknesses and tuned porous structures. By contrast, the main drawbacks are related to the use of a polymeric network embedding the adsorbent material and the possible redox reaction that could modify the properties of the supramolecular material.

## 3. Metal–Organic Frameworks

MOFs are crystalline, porous hybrid materials, based on the self-assembly of metal ions (or metal clusters) and organic ligands via coordinative bonds, leading to the development of 3D coordination polymers [7,17,25,29,31]. Their structure could be summarized considering the metals as the nodes of a highly porous crystal lattice, coordinated by rigid organic linkers, creating the scaffolds of the 3D framework. 

### 3.1. Synthetic Procedures

**Figure 3:**
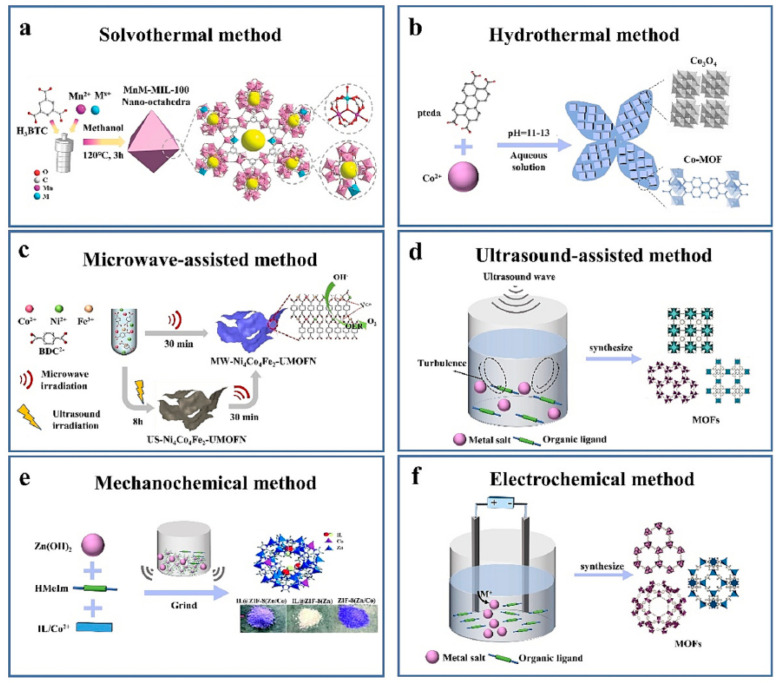
Schematic representation of the different approaches for MOF synthesis. (**a**) Solvothermal method; (**b**) hydrothermal method; (**c**) microwave-assisted synthesis; (**d**) ultrasound assisted method; (**e**) mechanochemical method; (**f**) electrochemical assisted approach. Reprinted with permission from Zhang et al. [38].

Different procedures have been proposed to synthesize MOFs, having a great impact on their structure and physical properties, namely morphology, lattice dimension, porosity, and surface area [31,38]. The main strategies used to produce MOF-based coatings rely on hydro/solvothermal methodology, microwave-assisted synthesis, sonochemistry, mechanochemical, and electrochemistry, as depicted in Figure 3.

Both the hydrothermal and the solvothermal approaches are based on the dissolution of metal salts and organic ligands in either water (hydrothermal) or an organic (solvothermal) medium, followed by reaction at high temperatures in pressurized reactors. The obtained material is then cooled, washed, and vacuum-dried to remove the solvent and sub-products from the pores of the framework. Although these approaches are simple, long reaction times and the use of high volumes of organic solvents or harsh conditions are usually required. Hydro/solvothermal synthesis has been frequently used for the in situ growth of MOFs [17,29,30,31,38]. To fasten the nucleation process, microwave-assisted synthesis has been proposed, obtaining MOF particles with a homogeneous size distribution in a very short time. Similarly, the ultrasound-assisted method has been proposed as a valid alternative to the previously described approach, being able to synthesize MOFs in shorter times, under milder conditions, and with high reaction yields. In fact, the acoustic cavitation provides a high local temperature and pressure, followed by high cooling rates, which facilitate both the reaction and the nucleation processes [38]. Microwave- and ultrasound-assisted synthesis are usually not compatible with in situ deposition; therefore, they are mostly proposed using the two-stage processes [31,38]. Recently, mechanochemical synthesis (MC), based on the use of a mechanical force to break the intramolecular bonds and promote chemical transformation, has emerged as a valuable green approach [39]. Being solvent-free, improvements in terms of reaction time, energy conversion efficiency, and reduction in organic waste, can be achieved. Finally, the electrochemical approach can be used to synthesize the MOFs directly onto the SPME fiber: the metal ions are obtained by anodic dissolution of the metal salts and react with the dissolved organic linkers.

### 3.2. MOF Features for SPME Extraction

Since their first application as SPME coatings in 2009 [40], MOFs have demonstrated excellent capability for the extraction of compounds belonging to different chemical classes due to their high surface area, good thermal and chemical stability, controlled pore size, and tunable surface chemistry by selecting metal ions, organic ligands, or synthetic conditions [25,29,31]. In particular, adsorption studies have demonstrated that after activation MOFs present surface areas characterized by Brunauer–Emmett–Teller (BET) values up to 7000 m^2^/g [29,31], higher than commonly applied SPME coatings such as activated carbon (~1500 m^2^/g), zeolites (300–600 m^2^/g), silica-based (300–800 m^2^/g) [41,42], or commercially available coatings, e.g., DVB (750 m^2^/g) [43]. The proper design of the MOF framework can also play a key role in enhancing selectivity by strengthening the steric complementarity between the host and the guests or by enhancing MOF–analyte interactions via hydrogen bonding, π-π, CH-π, electrostatic, van der Waals, hydrophobic/hydrophilic interactions, or via the coordination of the free metal sites present in the lattice [25,44]. 

### 3.3. MOFs as SPME Coatings for Environmental Applications

SPME based on MOF coatings has been widely applied for environmental monitoring, combining the simplicity, miniaturization, automation, and greenness of the extraction technique with the enhanced extraction capabilities and selectivity of 3D designed frameworks. These aspects are of paramount importance to meet the demand for analytical methods able to detect pollutants at trace levels in complex matrices [6,11]. The main application of MOF-based SPME coatings for environmental applications are discussed in the next subsections and summarized in Table 1.

#### 3.3.1. Extraction of Benzene, Toluene, Ethylbenzene, and Xylenes

Benzene, toluene, ethylbenzene, and xylenes (BTEX) are volatile non-polar organic compounds mostly derived from petrochemical fuel combustion and used as solvents in the pharmaceutical and dye industries. The International Agency for Research on Cancer (IARC) set benzene in group 1, ethylbenzene in group 2B, and toluene and xylenes in group 3. The main challenge in the detection of these compounds is related to their presence at trace levels in environmental samples, generally interfered with by the overwhelming amounts of aliphatic hydrocarbons [45,46]. Several MOFs have been proposed to improve the extraction efficiency and selectivity, including Zeolitic Imidazolate Frameworks (ZIFs) and Matériaux de l′Institut Lavoisier (MIL). These materials demonstrated both π-π stacking and size complementarity between the analytes and the framework. 

ZIFs are a class of MOFs that feature divalent metal nodes coordinated by imidazole organic ligands to obtain a material with a tetrahedron structure characterized by enhanced thermal and water stability. These materials have been applied as SPME coatings since 2010 [47]; however, in 2019 Maya and coworkers proposed the use of the single-crystal ordered macroporous (SOM) ZIF-8 as an SPME coating for BTEX extraction from wastewater samples [48]. The material was obtained by synthesizing a polystyrene monolith and a ZIF-8 MOF was grown in its interstices. Then, the polymer was dissolved using dimethylformamide, obtaining the SOM-ZIF-8 material, which was deposited onto SS using a silicone sealant. The obtained coating showed an ordered array of macropores and was characterized by 2.5–3.1-fold times higher intensities than both the crystalline ZIF-8 and the commercial PDMS fibers. The HS-SPME-GC-FID-based method was validated, obtaining limits of quantitation (LOQs) in the low ng/L level, allowing for BTEX detection in environmental samples. ZIF-8 was also the starting material for developing a superhydrophobic MOF composite material (_NS_ZIF-8^Si^) [49]: after synthesis of Mn_x_O_y_ nanosheets, in situ growth of a ZIF-8 MOF was performed on the nanomaterial, followed by deposition of PDMS on its surface. The SPME fiber coating was obtained by physical adhesion using a silicone sealant. The developed material showed excellent thermal stability (up to 450 °C), superhydrophobic behavior, and significantly higher extraction efficiencies than commercial 30 μm PDMS due to the combined hydrophobic and π-π stacking effects. 

Recently, Hu and coworkers [50] used the MIL-101-NH_2_ framework, a moisture-sensitive MOF, as a template to develop a carbonaceous coating. After solvothermal synthesis, cobalt and thiourea were encapsulated inside the MOF structure and the material was annealed, obtaining urchin-like nanoporous carbon. The final coating was obtained by physical deposition using a silicone sealant. The extraction performance of the urchin-like nanoporous carbon-based SPME coating was better than that obtained by using commercial PDMS and MIL-derived nanoporous carbon not functionalized with cobalt and thiourea, achieving LOQ values in the 0.28–1.2 ng/L range. Finally, the material provided a very high sampling rate for all the analytes, reaching extraction equilibrium within 2 min. Kardani and coworkers developed a polyacrylonitrile/MIL-53(Al)MOF@SBA-15/4,4′-bipyridine hybrid nanocomposite [51]. The material was based on mesoporous silica obtained by the sol–gel procedure, functionalized with a MIL-53(Al) MOF by solvothermal reaction. The coating was obtained by electrospinning PAN onto an SS fiber in the presence of the hybrid nanocomposite material. A very porous structure with a high degree of cross-linking and increased adsorption/desorption rates was achieved. Finally, LOQs in the 7.6–20 ng/L range allowed the ultra-trace determination of BTEX in wastewater samples. 

BTEX extraction from air samples using a MOF-199-based coating deposited in situ via a solvothermal approach was proposed by Omarova et al. [52]. Different thicknesses of the MOF-199 coating were tested for the extraction of 20 low-molecular-weight VOCs including BTEX and 3 high-molecular-weight VOCs. For most of the substituted monoaromatic compounds, the extraction capabilities of the developed coating were significantly higher than those achieved by using a PDMS/DVB fiber. Finally, LOQs in the range of 0.09–0.31 μg/m^3^ proved the reliability of the MOF-199 coating for air monitoring purposes. 

#### 3.3.2. Extraction of Polycyclic Aromatic Hydrocarbons

Polycyclic aromatic hydrocarbons (PAHs) are a broad class of compounds consisting of two or more fused aromatic rings, exhibiting a linear, angular, or cluster arrangement. These compounds are characterized by extremely low water solubility, very different vapor pressures, high toxicity, and high bioaccumulation potential; therefore, they are considered as priority pollutants by both the United States Environmental Protection Agency (US EPA) and the European Environment Agency [53]. The main analytical challenge is related to their detection at sub-trace levels in the presence of overwhelming amounts of interfering compounds.

ZIF-based coatings were developed and tested for the extraction of PAHs using different deposition approaches or post-synthetic functionalization of the MOFs. In 2019, Kong and coworkers synthesized ZIF-8 MOFs by different approaches, namely solvothermal, stirring, and ball-milling methods, and deposited the MOF-based material onto an SS wire using a dual-stage deposition via the sol–gel technique [54]. The obtained coatings were tested for the direct immersion (DI)—SPME—gas chromatography—mass spectrometry (GC—MS) analysis of 16 US EPA PAHs and 11 nitro-PAHs. The best enrichment capabilities were obtained by using the solvothermally synthesized material, which was also characterized by the highest BET surface area (1390 m^2^/g). Under optimal conditions, LOQs in the 1.1–90.0 ng/L range were obtained, thus assessing the reliability of the method for the detection of PAHs at sub-trace levels. Finally, the analysis of environmental samples highlighted high levels of contamination of river and lake water and industrial wastewater, with PAHs detected in the 30.0(±1.1)–1509(±83) ng/L range. A ZIF-8-based SPME coating was synthetized in situ onto metal alloy wires by CVD and then solvothermally grown by Rocio-Bautista and coworkers [55]. The coating was applied for the extraction of acenaphthene, fluorene, and pyrene as model PAHs together with homosalate, ethylhexylsalicilate, methyl-antralinate, padimate-O, and ethylhexyl 4-methoxycinnamate as personal care product representatives. The DI-SPME-GC-FID method was characterized by limit of detections (LODs) in the 0.6–2 μg/L range and relative standard deviations (RSDs) ≤ 23%; however, a strong matrix effect was observed when wastewater samples were analyzed. Lian and coworkers studied the in situ electrochemical deposition of ZIF-8 onto SS wires pretreated using dopamine under alkaline conditions [56]. A homogeneous porous coating characterized by very high thermal stability (up to 460 °C) was obtained. The coating was tested for the HS-SPME-GC-FID analysis of eight low-molecular-weight PAHs obtaining LOQs in the 35–179 ng/L range, allowing for the detection of acenaphthene, acenaphthylene, and fluoranthene in lake water at concentration levels in the 200 ± 13–360 ± 33 ng/L range. Zeng et al. developed ZIF-8-based coatings with tunable properties in terms of polarity, porosity, surface area, and conductivity, thus allowing the extraction of either polar or apolar compounds [57]. SS wires coated with PANI deposited by means of cyclic voltammetry were used as an SPME support. Aligned ZnO nanorods were deposited using a dipping -seed-mediated method and then a hydrothermal method was applied for ZIF-8 in situ growth. The developed coating was tested both for the electroenhanced SPME extraction (EE-SPME) of aromatic amines and ionic drugs in spiked standards and for the DI-SPME-GC-FID analysis of six low-molecular-weight PAHs in sewage water samples. Electrochemical deposition was applied for a two-step synthesis to obtain a nickel/titanium alloy coated with cobalt and for the in situ growth of a ZIF-67 coating [58]. The material was then annealed to obtain a Co@ZIF- 67-derived coating to be used for the selective DI—SPME—liquid chromatography—UV detection (LC—UV) of six PAHs. The proposed material was characterized by a higher extraction capability and selectivity than commercially available materials and other developed coatings, with LODs in the 5–42 ng/L range, good linearity, and precision (RSD ≤ 7%). Finally, the method was applied for the analysis of snow, lake and river water, and wastewater samples, detecting a total concentration (Σ) of six PAHs in the 1.47–6.34 μg/L range.

Metal azolate frameworks (MAFs) were also tested as SPME coatings by Liu et al. [59] and Wang et al. [60] for the extraction of 7 and 16 PAHs in water, respectively. In both studies, Zn was used as the metal ion and the coatings were obtained by a two-stage methodology consisting of a preliminary step based on the solvothermal synthesis of the MOFs, followed by the dipping of etched SS fibers in a methanolic solution containing the dispersed MOFs. In Liu et al. [59], the synthesis of MAF-66 was performed at room temperature and the layer-by-layer deposition was completed after 10 dipping cycles. The SPME fiber was then used for the HS-SPME-GC-FID analysis of low-molecular-weight PAHs, obtaining LODs in the 0.1–7.5 ng/L range. Two different materials, namely MAF-5 and MAF-6, were tested by Wang et al. [60] for the extraction of the 16 US EPA priority pollutant PAHs from aqueous samples, showing that MAF-6 had the highest extraction capability. Finally, the DI-SPME-high-performance liquid chromatography-fluorescence detection (HPLC-FLD) method was characterized by LOQs at the sub-μg/L level for all the investigated PAHs, except for fluoranthene (LOQ 1.8 μg/L) and benzo(k)fluoranthene (LOQ 1.5 μg/L). 

Sun et al. developed a HKUST-1-based SPME coating using both the in situ growth and the two-stage process using neutral silicone gel as the adhesive [61]. Both coatings were tested for the HS-SPME-GC-MS analysis of eight PAHs from aqueous samples: the in situ grown coating provided a better extraction capability compared to the two-stage coating due to the pore size and radial alignment of channels. In addition, in situ growth is a less time-consuming procedure. The method was characterized by LOQs in the 0.4–33 ng/L range and proved to be suitable for the extraction of eight PAHs from lake water samples, allowing the detection of medium-weight PAHs in both sites, with concentrations in the 15.3–64.4 ng/L and 19.6–56.9 ng/L range, respectively.

Bianchi et al. tested the extraction capability of a triple-catenated Zn^2+^-pillared MOF, named PUM-210, for the extraction of 16 US EPA PAHs [62]. Compared to the commonly applied homoleptic materials, this framework incorporates two different ligands, i.e., 2,6-naphthalenedicarboxylic acid and a pyridine-functionalized biphenylene ligand, for the complexation of Zn(II) ions. The MOF was obtained by solvothermal synthesis and the coating was deposited onto a silica fiber by means of epoxy glue. The developed coating provided low quantitation limits, in the 1–7 ng/L range, and very high enhancement factors in the 300–14,950 range. Finally, the addition of BTEX as interfering compounds did not affect the quantitation of PAHs due to the presence of additional π–π interactions, higher hydrophobicity, and size complementarity between the MOF and the analytes. 

**Figure 4:**
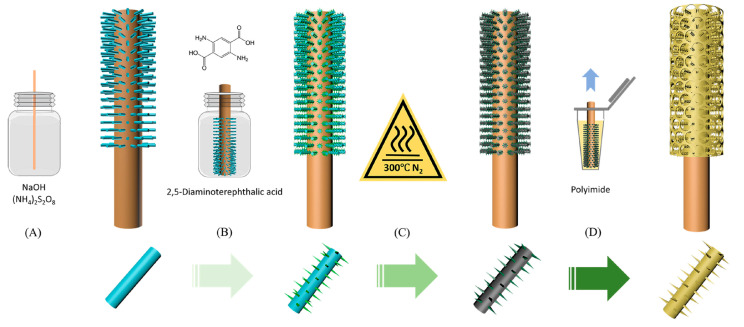
Schematic representation of the in situ heteroepitaxial growth of copper-2,5-diaminoterephthalate crystals on copper wires, followed by dip-coating: (**A**) immersion of copper wire in the conversion solution; (**B**) immersion of the treated copper wire in the saturated ligand solution; (**C**) coating annealing; (**D**) dip-coating using polymide solution. Reprinted with permission from [63].

Qiu et al. developed a sheathed MOF-based SPME coating through the in situ heteroepitaxial growth of copper-2,5-diaminoterephthalate crystals (Cu-DAT) on copper wires, followed by dip-coating to obtain a PI sheath [63]. The coating was deposited by a multi-step synthesis directly onto the copper wire (Figure 4): (A) copper hydroxide nanotubes were grown onto the fiber to provide a substrate for the in situ growth of the MOF; (B) the fiber was immersed in the ligand solution; (C) the material was annealed; and (D) the PI sheath coating was obtained by dip-coating. The obtained fibers were tested for the extraction of 10 model PAHs from both water samples and fish muscle by using both HS and DI extraction, obtaining LOQs in the range of 0.3–2.1 ng/L and 4.0–18.9 ng/g, respectively. Finally, the reliability of the developed procedure was assessed both for the detection of PAHs in river water and for their in vivo monitoring in tilapia dorsal muscle, obtaining results comparable to conventional liquid extraction.

#### 3.3.3. Extraction of Organophosphorus and Organochlorine Pesticides

Organophosphorus (OPP) and organochlorine (OCP) pesticides are widely employed in agriculture for pest control. Being toxic and persistent compounds, they can exert a harmful impact on both ecosystems and human health, thus requiring strict monitoring to guarantee compliance with the current regulation [64]. To face this challenge, selective, sensitive, and reliable SPME methods based on MOF coatings have been proposed [65,66,67].

Electrospinning was applied by Amini and coworkers to deposit a polyacrylonitrile/nickel-based MOF (PAN/Ni-MOF) nanocomposite coating on an SS wire for the extraction of diazinon and chlorpyrifos from environmental water samples [65]. The obtained hydrophobic coating exploited the capability of the nitrile backbone of PAN to promote hydrogen bonding and π-π interactions with the OPPs. Corona discharge-ion mobility spectrometry (CD-IMS) was applied for the detection of diazinon and chlorpyrifos, obtaining LOQs of 0.5 and 1 μg/L, respectively. Finally, the reliability of the developed method was assessed by analyzing river water (no pesticide detected), agricultural wastewater (Σpesticides 24.6 μg/L), and groundwater samples near rice fields (Σpesticides 20.0 μg/L).

Mohammadi et al. developed a flexible/self-supported ZIF-67 film for thin-film microextraction of OPPs from agricultural wastewater and underground water [66]. Firstly, electrospinning followed by calcination was used to obtain cobalt oxide fibers; in a second step, functionalization with the MOF was carried out by hydrothermal reaction. The developed film was used for the extraction of ethion using secondary electrospray ionization–ion mobility spectrometry (SESI-IMS) as a detection technique, requiring only 2 min of detection time per sample. In addition, the thermal desorption of the film was in accordance with the principles of analytical green chemistry. Under optimized conditions, a LOQ of 500 ng/L was obtained, allowing for the detection of ethion in one agricultural wastewater sample (1.1 μg/L).

A novel MOF coating was developed by Gong et al. using a two-stage deposition process: a NU-1000 MOF was synthesized by solvothermal reaction and deposited onto SS wires using a silicone sealant [67]. The major features of the framework were the very high surface area (BET 1966 m^2^/g) and the presence of mesoporous channels able to increase mass transfer inside the coating. The material was tested for the extraction of six model OCPs at ultra-trace levels from water samples. Although the enrichment factors (EFs) were higher than those obtained by using either commercial 65 μm PDMS/DVB or 85 μm PA fibers were achieved, selectivity issues were observed. In fact, when PAH extraction was carried out, a high adsorption capability was observed, with EFs in the 9800–11,000 range, due to the π-π and CH-π interactions between the polyaromatic compounds and the coating. Under optimized conditions, LOQs in the range of 37–190 ng/L were obtained; thus, the DI-SPME-GC-MS method was suitable for ultra-trace analysis of OCPs in river and pond water, detecting only hexachlorobenzene at a concentration of 1.86 ± 0.08 ng/L and 2.08 ± 0.13 ng/L, respectively.

Xu et al. developed a lotus-like Ni@NiO–embedded porous carbon (Ni@NiO/PC) SPME coating for the extraction of chlorobenzenes from aqueous samples [68]. The coating was prepared following a two-stage deposition process: after solvo- and hydrothermal synthesis of cellulose nanocrystals functionalized with MOF-74, the material was pyrolyzed to obtain the Ni@NiO/PCs, which was deposited onto SS wires by means of a silicone sealant. The obtained coating was able to adsorb the chlorobenzenes more effectively compared to both the pristine materials and commercial coatings. The target analytes could diffuse through the surface of the material due to the presence of meso- and micropores with a size of 1.26 nm, interacting with the active sites of the coating via dipolar (thanks to the Ni-O groups) and π-π and hydrophobic interactions. The coating was tested for the DI-SPME-GC-MS analysis of eight model chlorobenzenes in tap and surface water: despite the LOQs ranging from 0.017 to 0.118 ng/L, no chlorobenzene was detected in tap water, whereas 1,3,5-trichloro-benzene (15.7, 28.9 ng/L), 1,2,3-trichloro-benzene (14.4, 10.9 ng/L), and 1,2,4,5-tetrachloro-benzene (1.4 ng/L) were detected in river water samples.

#### 3.3.4. Extraction of Poly- and Perfluoroalkyl Substances

Poly- and perfluoroalkyl substances (PFASs) are a wide class of emerging pollutants, accounting for thousands of synthetic compounds with linear, branched, or cyclic structures, characterized by a hydrophilic terminal, such as a carboxylic or sulfonate group, and a hydrophobic fluorinated alkyl chain. PFASs have been widely used in manufacturing, packaging, the textile and semiconductor industry, or as foams and detergents [69]. Owing to the chemical and biological stability of the C-F bonds, these compounds are extremely persistent in the environment and can be easily accumulated in the tissues of living organisms, thus being considered as a major threat to human life and ecosystems [69]. Both EU and US EPA have set stringent regulations for PFASs, limiting their concentration at trace levels in drinking water and environmental samples. Analytical monitoring of these compounds is a challenging issue due to their unique hydrophobic and oleophobic nature [69]. In this context, MOFs have been proposed as valuable coating materials for PFAS extraction [70,71,72,73].

Four different water-resistant MOFs, namely, ZIF-8, UiO-66, MIL88-A, and Tb_2_(BDC)_3_, were investigated as coatings for probe extraction of perfluorooctanoic acid (PFOA) from environmental water, being that this compound is one of the most widely spread PFASs [70]. The coating was obtained by in situ growth of the MOFs onto poly(dopamine) (PDA)-coated SS needles. ZIF-8 and UiO-66 were able to adsorb PFOA inside the pores and exhibited similar binding energies, whereas MIL88-A and Tb_2_(BDC)_3_ ZIF-8 were characterized by surface adsorption, being that their pores were too small for hosting the analyte. Due to the lower production costs and higher reproducibility, only ZIF-8 was selected for PFOA quantitation. Finally, a DI-SPME extraction followed by direct analysis by nanoelectrospray ionization–mass spectrometry (nanoESI-MS) method was validated, allowing for very rapid analysis (10 min per sample, including pretreatment and MS analysis) and achieving a LOD value of 11 ng/L. Therefore, the reliability of the method was demonstrated for the purpose of high-throughput screening of PFOA at trace levels. The validated method was applied for the quantitation of PFOA in contaminated samples of tap water, rainwater, and seawater. A strong matrix effect was observed, especially when seawater samples were analyzed, obtaining a sensitivity (7.75 × 10^8^ M^−1^) higher than that observed in ultrapure water (2.43 × 10^8^ M^−1^), probably as a consequence of the alkalinity and ion abundance of seawater.

**Figure 5:**
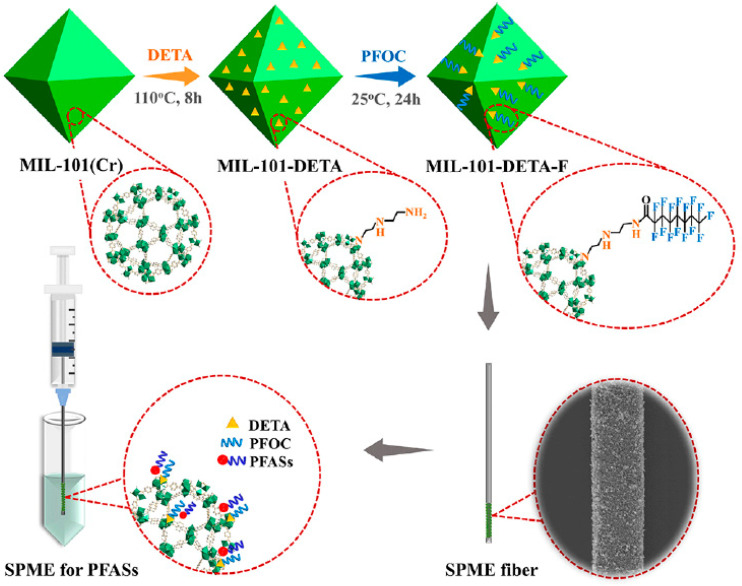
Schematic representation of the synthesis and deposition of dual-functionalized MIL-101-DETA-F onto SPME fibers, reprinted with permission from [73].

Another strategy to improve the enrichment capability of SPME through electrostatic interactions and hydrogen bond formation between the amine group and the acidic group of PFAAs was based on the functionalization of MOFs with amino groups [71,72,73]. In this context, the extraction of eight model PFASs using an amino-functionalized ZIF-8 (NH_2_-ZIF-8) coating followed by HPLC-MS/MS analysis was proposed by Gong and coworkers [71]. The MOF was synthesized by solvothermal reaction and deposited onto quartz fibers by means of PAN. The developed coating showed a higher performance than pristine ZIF-8 and commercial fibers, namely 65 μm PDMS/DVB and 85 μm PA fibers. After optimization and validation, the DI-SPME-HPLC-MS/MS method was applied for the analysis of river water, seawater, and wastewater treatment plant effluent samples, allowing for the detection of the target analytes at ng/L levels. In 2022, Ouyang et al. proposed a modification of UiO-66(Zr) introducing both amino groups on the surface and the end-capping of the material to avoid any interaction with water molecules, thus enhancing the hydrophobic interactions [72]. The NH_2_-UiO-66(Zr) MOF was obtained in a one-step solvothermal synthesis, capped using phenylsilane, and deposited onto the fiber by means of PAN. The presence of capped nanocrystals (NH_2_-UiO-66(Zr)-hp) resulted in higher hydrophobicity, faster extraction kinetics and higher enrichment capacity compared to the uncapped coating. The DI-SPME-HPLC-MS/MS method was validated, obtaining LOQs in the range of 0.21–1.8 ng/L, and applied for the analysis of tap, river, and pond water samples detecting ΣPFASs of 29.3, 58.7, and 45.0 ng/L, respectively. A similar approach was proposed by Jia et al., who developed an SPME coating based on MIL-101(Cr) functionalized with amino and fluorinated groups to enhance the interaction towards PFASs [73]. As depicted in Figure 5, the bifunctional MOF was synthesized by the hydrothermal method, aminated by diethylenetriamine (DETA), and fluorinated using perfluorooctanoyl chloride. Finally, the coating was obtained by dipping the SS needle coated with epoxy resin in the developed material. The dual-functionalized MIL-101(Cr) (MIL-101-DETA-F) SPME coating was applied for the extraction of nine model PFASs, obtaining EFs in the 70–112 range: these values were higher than those obtained by using UiO-66(Zr), MIL-101(Cr), the mono-amino-functionalized MOF, and the commercial PDMS/DVB coatings. In addition, the pore size of MIL-101-DETA-F was close to the molecular size of perfluorododecanoic acid, allowing for the size exclusion of interfering compounds. Compared to the monofunctionalized MOF, fluorination enabled F−F interactions, improving both the selectivity and extraction capability of SPME. Finally, the DI-SPME-ultra-high-performance liquid chromatography-tandem mass spectrometry (UHPLC-MS/MS) method was validated for PFAS extraction in aqueous samples, achieving extremely low LOQ values, in the range of 0.01−0.40 ng/L, a three order of magnitude linear range, and RSDs ≤ 12%. The ΣPFASs was 5.5 and 27.2 ng/L for tap and river water, respectively, and in the 263.8–2790.7 ng/L range for municipal, dyeing, and mining wastewater samples.

#### 3.3.5. Extraction of Pharmaceutical and Personal Care Products

Pharmaceutical and personal care products (PPCPs) are emerging pollutants with potential detrimental effects on human health, the environment, and ecosystems. Their widespread use, inappropriate disposal, and incomplete removal by conventional wastewater treatment procedures have led to their ubiquitous presence in all the environmental compartments. Due to their presence at trace or sub-trace levels in various environmental matrices and their different physical and chemical properties, the development of a reliable method for their monitoring is still a challenging issue [74].

In this context, new MOF-based coatings for the detection of nonsteroidal anti-inflammatory drugs and anti-cancer drug residues have been reported by the research group of Liu and Khodayari [75,76]. In particular, a zirconium-based MOF and graphene oxide nanocomposite coating (Zr-MOF@GO) was synthesized by hydrothermal reaction and deposited onto SS wires by means of an aluminum chromium phosphate binder and applied for the DI-SPME-GC-FID determination of ibuprofen and diclofenac [75]. The extraction mechanism of the hybrid material was based on free-metal site coordination, hydrogen bond formation, and π–π interactions, achieving LOQs of 10 and 100 ng/L, respectively. Finally, the method was applied for the quantitation of the investigated drug residues in a Yellow River water sample, obtaining similar results to those achieved using a commercial PA fiber, i.e., an ibuprofen concentration of 27 ng/L and a diclofenac concentration below the LOQ. Khodayari and coworkers [76] developed electrospun polyfam/Co-MOF-74 composite nanofibers for the thin-film SPME extraction of sorafenib, dasatinib, and erlotinib, used as anti-cancer drug representatives. The Co-MOF-74 particles were obtained by the hydrothermal procedure and deposited onto the SS fibers by electrodeposition using polyfam as a conductive polymer. The incorporation of the MOF within the polyfam fiber network produced a significant increase in both the surface area and total pore volume compared to the pure polyfam electrospun fibers (28.3 m^2^/g and 0.244 cm^3^/g vs. 8.4 m^2^/g and 0.044 cm^3^/g), thus improving the extraction efficiency toward the anti-cancer drugs. The optimized thin-film-SPME-HPLC-UV method was validated, obtaining EFs in the 24.2–37.1 range and allowing for the quantitation of the analytes at the sub-μg/L level. Finally, the analysis of environmental water samples revealed a high level of contamination, being that the concentration levels of the detected analytes were always higher than 10 μg/L.

Linear and cyclic methylsiloxanes are another class of contaminants of emerging concern, being used in the formulation of PPCPs to improve their physical properties and as precursors of silicone polymers. To improve the detection of these pollutants in wastewater samples, both a MIL-101-based and a CIM-80(Al)-based coating were recently proposed by Zhang et al. and by González-Hernández and coworkers [77,78]. The MIL-101-based fiber was obtained by SS fiber dip-coating in a solution containing MIL-101 and polysulfone [77] and an HS-SPME-GC-MS method was validated for the extraction of octamethylcyclotetrasiloxane, decamethylcyclopentasiloxane, and dodecylcyclohexasiloxane from environmental water samples. Under optimal conditions, the MIL-101-based method provided LOQs in the 140–270 ng/L range and RSDs ≤ 4%. The extraction performance of the developed coating was better or equal to those obtained by using a commercial 65 μm PDMS/DVB fiber, which was affected by high background contamination. Finally, the validated method was applied to quantify the target analytes in wastewater samples collected at different treatment stages from a wastewater treatment plant in Guangzhou, China, obtaining the highest contamination levels in the aeration unit (1.8–85.1 μg/L) and barscreen (0.7–3.3 ng/L). Regarding the CIM-80(Al)-based coating, it was obtained on APTES-pretreated nitinol wires by in situ growth [78]. The fibers were used for the HS-SPME-GC-MS analysis of six linear and cyclic methylsiloxanes and seven musk fragrances as model PCPs. The performance of the MOF-based coating was compared with that achieved by using the commercially available 65 μm PDMS/DVB-coated fibers: the developed coating provided lower or comparable LOQs for cyclic methylsiloxanes compared to the commercial one (in the 0.2–0.7 μg/L vs. 0.5–0.6 μg/L range), whereas an opposite behavior was observed for linear methylsiloxanes (0.1–0.4 μg/L vs. 0.05–0.06 μg/L). As for musk fragrances, LOQs in the 1.2–3.5 μg/L and 0.1–0.4 μg/L ranges were obtained for the MOF-based and commercial coating, respectively. However, the CIM-80(Al)-based coating demonstrated slightly better precision, a larger linear range, and reduced cross-contamination compared to the commercial fibers. Finally, the HS-SPME-GC-MS method was applied for the analysis of three seawater and three wastewater samples, detecting cashmeran (15 ± 2 and 1.4 ± 0.2 μg/L) and galaxolide (46.9 ± 0.2 and 6 ± 1 μg/L) in two out of three wastewater samples, whereas no analyte could be quantified in the analyzed seawater samples.

A superhydrophobic amino-functionalized UiO-66(Zr) coating was proposed by Liu et al. for the extraction of semi-volatile UV filters from environmental water samples [79]. The superhydrophobic MOF was synthesized in solvothermal conditions and deposited onto SS wires by means of a neutral silicone sealant. By capping the NH_2_-UiO-66(Zr) MOF with phenylsilane groups, the adsorption of water molecules within the MOF lattice was strongly reduced, thus increasing the enrichment capability of the coating up to six and eight times higher compared to PDMS and unfunctionalized MOFs, respectively. After optimization and validation, the HS-SPME-GC-MS method, characterized by LOQs in the range of 0.7–7.1 ng/L and RSDs ≤ 6%, was applied for the quantitation of UV filters in river and pond water samples, detecting concentration levels in the 40.1–193.7 ng/L and 26.1–250.1 ng/L ranges, respectively.

A ZIF-8-based coating was developed for the in-tube (IT)-SPME-HPLC-FLD detection of five model fluoroquinolones in tap water, river water, and wastewater samples [80]. Firstly, a porous monolith was obtained by copolymerization of 4-vinylbenzoic acid with ethylenedimethacrylate in a fused silica capillary. Then, the ZIF-8 MOF was obtained by in situ layer-by-layer deposition using ZnNO_3_ and imidazole solutions, allowing both a controlled self-assembly of the MOF and a good column-to-column reproducibility (RSDs < 10%). The IT extraction allowed for the on-line SPME-HPLC-FLD analysis of the aqueous solutions containing the analytes, obtaining LOQs in the 0.48–1.8 ng/L range and EFs in the 255–296 range. Finally, the reliability of the method was assessed by detecting low concentration levels of fleroxacin, enrofloxacin, and sarafloxacin in the investigated samples.

#### 3.3.6. Extraction of Polychlorinated Biphenyls

Polychlorinated biphenyls (PCBs) are a class of persistent organic pollutants accounting for more than 200 synthetic organic compounds. PCBs are listed among the top five priority hazardous substances by the US EPA due to their toxicity, persistency, ubiquitous presence, and bioaccumulation [81]. Despite their production being banned in the 1970s, they have been detected in all the environmental compartments and ecosystems, including air, water, soil, and in wildlife, plants, animal tissues, and food products. Considering that PCBs are listed by the IARC in group 1 (carcinogenic to humans) and are recognized endocrine disruptors, their monitoring in the environment is of pivotal importance: to this aim, different MOF-based SPME coatings have been devised [82,83,84]. 

A coating with nitrogen-doped carbon nanotube cages (N-CNTCs), derived from ZIF-67 obtained by calcination of the MOF and deposited onto SS wires by means of an epoxy resin, was successfully proposed for PCB determination [83]. The MOF morphology was preserved, obtaining a thin N-doped carbon nanotube assembled structure with uniform hollow cages. The material showed better extraction performance than that achieved using either commercial coatings (75 μm CAR/PDMS and 65 μm PDMS/DVB fibers) or the non-hollow material, which also required prolonged time to reach equilibrium. The extraction capability of the N-CNTCs was attributed to π-π interactions between the analytes and the interconnected graphitic framework, as well as to the N doping. The DI-SPME-GC-MS method was characterized by LOQs in the 0.33–0.72 ng/L range, a four order of magnitude linear range, and good precision. The method was applied for the determination of PCBs in six surface water samples, quantifying PCBs in the 1.9–26.2 ng/L range. Similarly, hollow carbon nanobubbles (HCNBs) were obtained from a ZIF-8 MOF [84]. The developed material maintained the morphology of the framework, also featuring an internal hollow structure able to provide short diffusion distances and increased extraction capability. The SPME coating was tested for the extraction of different classes of persistent pollutants, namely BTEX, PAHs, and PCBs, always showing higher extraction capabilities than commercial PDMS and PDMS/DVB fibers. As in Guo et al. [83], the presence of a high content of nitrogen in the structure increased the adsorption sites and surface polarity, resulting in the efficient extraction of PCBs. The HS-SPME-GC-MS method was optimized and validated, obtaining LOQs in the 0.0017–0.0042 ng/L range, a five order of magnitude linear range, and RSDs ≤ 14%. Finally, the method was tested for the extraction of five model PCBs from rainwater, pond, and river water samples, obtaining ΣPCBs of 37.1, 64.1, and 100.2 ng/L, respectively.

#### 3.3.7. Extraction of Other Compound Classes

A hollow zirconium–porphyrin-based MOF (HZ-PMOF) was proposed as an SPME coating for the extraction of 1-naphthol and 2-naphthol, which are extremely toxic environmental contaminants, widely used in industry as precursors for the production of dyes, pesticides, and pharmaceuticals [85]. The MOF was synthetized by a solvothermal approach and physically deposited by means of a polyimide sealing resin. Comparing the non-hollow with the hollow MOF material, different morphologies were obtained: the HZ-PMOF exhibited mesopores and a BET surface of 1585 m^2^/g, with benefits in terms of mass transfer efficiency and specific surface area, whereas the non-hollow material was characterized only by micropores and a surface of 997 m^2^/g. The developed HS-SPME-GC-MS/MS method based on the HZ-PMOF-coated fiber was validated, obtaining low LODs (1.0 ng/L) for both napthols and, applied to the analysis of environmental water samples from five cities in China, detecting the analytes in two out of five samples (37.7 ± 5.6 and 15.0 ± 3.4 ng/L for 1- naphthol and 48.9 ± 4.3 and 8.9 ± 2.1 ng/L for 2- naphthol, respectively).

Darabi and Ghiasvand developed a PPy/chromium-based MOF nanocomposite, PPy@MIL-101(Cr), which was obtained by the hydrothermal method and deposited by electrochemical polymerization of pyrrole onto SS wires [86]. The coating was tested for the extraction from soil samples of methyl tert-butyl ether, a persistent environmental contaminant used as an octane enhancer in gasoline [87]. The novel material showed a higher extraction capability than commercially available materials and pure electrodeposited PPy due to the larger sorptive surface area and stronger interactions. The validation of the HS-SPME-GC-FID method demonstrated a LOQ value of 0.5 ng/g and a linear response over four orders of magnitude. As a main drawback, a low intra-fiber reproducibility was observed, with RSDs up to 26% due to the scarce robustness of the electropolymerization process. Finally, the validated method was applied for the quantitation of methyl tert-butyl ether in six soil samples collected from an oil refining company and different gas stations, detecting the analyte in the 0.92(±0.48)–2.72(±0.50) μg/g range.

In 2019, Wei et al. developed a porous carbon SPME coating performing both hydrothermal growth of MOF-74 and in situ carbonization on SS wires [88]. The MOF-74-C coating was tested for the extraction of five odorous organic contaminants, namely, 2-chlorophenol, 2,4,6-trichloroanisole, 2-isobutyl-3-methoxypyrazine, thiophenol, and 4-methylthiophenol from water samples. The developed material showed an extraction efficiency 2.0–16.0 times higher than PDMS and PA fibers, whereas similar results were obtained using the PDMS-DVB fiber. Compared to the commercial polymeric materials, the MOF-74C coating was characterized by enhanced selectivity due to the presence of micropores with sizes ranging from 0.59 to 1.71 nm, very close to the dynamic diameters of the odorants, thus resulting in a sieve and micropore-filling effect. The DI-SPME-GC-MS method was validated, obtaining LOQs in the 0.03–300 ng/L range, a three-to-five order of magnitude linear range, recoveries in the 90.1–107.3% range, and RDSs always lower than 8.8%. The method was finally applied to the analysis of wastewater, tap, and river water samples, detecting 2-isobutyl-3-methoxypyrazine in the surface water sample at trace levels (0.2 ng/L).

**Figure 6:**
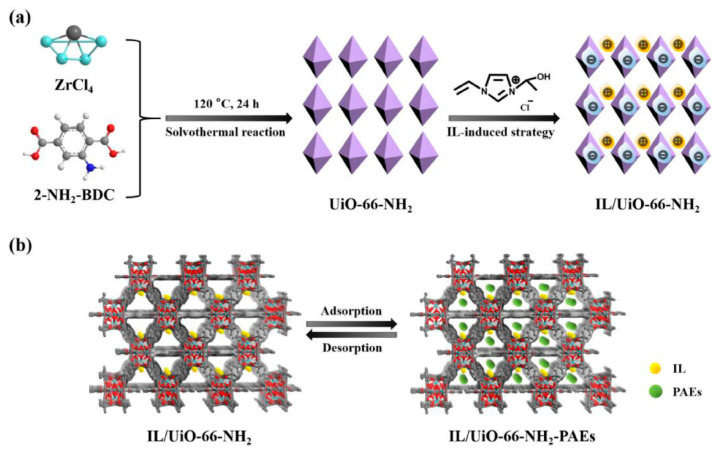
Schematic of the fabrication of IL/UiO-66-NH 2 (**a**) and the extraction and desorption process for PAEs: (**a**) fabrication of the coating; (**b**) extraction/desorption process for PAEs. Reprinted with permission from [90].

UiO-66-NH_2_ can be considered as an excellent MOF platform to obtain hybrid materials [89,90,91]. In 2019, Ni et al. developed zirconium and nitrogen co-doped ordered mesoporous carbon (Zr/N-OMC) SPME coatings to be used for the extraction of substituted phenolic compounds [89]. UiO-66-NH_2_ was obtained by solvothermal synthesis, dispersed in a solution containing phenolic resin precursors, and the composite resin/UiO-66-NH_2_ material was synthesized by solvent-evaporation-induced self-assembly. Finally, Zr/N-OMC was obtained by carbonization of the composite and deposited onto SS wires using a neutral silicone sealant. Zr/N-OMC was characterized by faster equilibrium rates and a higher extraction efficiency compared to non-doped and N-doped OMC, despite the similar pore structure and lower BET surface (775, 720, and 583 m^2^/g for OMC, N-OMC, and Zr/N-OMC, respectively). Zr doping increased the adsorption rates and the extraction amounts of phenols, benefiting from Lewis acid–base interactions between the coating and the analytes. The hybrid coating was applied for the extraction of six model phenols from water samples and the HS-SPME-GC-MS method was validated, achieving LODs in the 0.21–1.7 ng/L range, a three order of magnitude linear range, and a fiber-to-fiber precision with RSDs ≤ 10%. The method was applied for the extraction of phenols from surface water: all the analytes except 2,4,6-trichlorophenol were detected in the 20.0(±1.0)–315.0(±10.0) and 20.0(±1.2)–330(±12.0) ranges in river and pond samples, respectively. UiO-66-NH_2_ was also used to develop a new SPME coating that combines the extraction capability of MOF with ionic liquids (IL) [90]. The coating was deposited by treating the SS fibers with hydrofluoric acid and hydrogen peroxide to obtain hydroxyl groups for in situ MOF growth. The functionalization with the 1-hydroxyethyl-3-vinylimidazole chloride was performed in situ, exploiting the interaction between the anion and the zirconium nodes (Figure 6); the final IL/MOF was used for the DI-SPME extraction of phthalate esters (PAEs) from water samples. Interestingly, the incorporation of the IL inside the MOF increased the hydrophobicity of the channels while promoting hydrogen bond formation and π-π interactions between the coating and the PAEs. The developed coating provided a higher extraction efficiency than commercial 100 μm PDMS, 75 μm CAR/PDMS, and 65 μm PDMS/DVB fibers, and the non-ionic coating. Under optimal conditions, the validated DI-SPME-GC-MS method was showed by LOQs in the 0.6–1.2 ng/L range, a three order of magnitude linearity, and precision, with RSDs ≤ 12%. Finally, the method was tested for the extraction of PAEs from spiked surface and bottled water samples, obtaining good recoveries in the 90.3–102.4% range.

Finally, an interesting study was carried out by Xu and coworkers, who developed an aptamer-functionalized MOF (PAN/UiO@UiO2-N3-aptamer), based on the UiO-66-NH_2_ structure [91], for the selective LC-MS recognition of Microcystin-LR at trace levels. The UiO-66-NH_2_ seed crystals were prepared using solvothermal conditions and electrospun with PAN to obtain a PAN/UiO SPME coating. The MOF was grown in situ by a seeding procedure and functionalized to obtain the azide intermediate to be used as binding site for the aptamer. The specific affinity recognition of the material toward the target analyte was demonstrated by comparing the performance of the PAN/UiO@UiO2-N3-aptamer with that of the intermediate materials and the MOF functionalized with a control oligonucleotide for the extraction of Microcystin-LR in the presence of two different structural analogs. The results highlighted the superior performance of the PAN/UiO@UiO2-N3-aptamer coating, characterized by a high extraction capability, selectivity, and recovery up to 95.2%. By contrast, no significant difference in terms of adsorption capability was observed when the other materials were used, thus indicating that non-specific interactions were responsible for the adsorption of the compounds. The DI-SPME-LC-MS method was validated, obtaining a LOQ of 8 ng/L, a three order of magnitude linear range, and good precision (RSDs < 13%). Finally, the method was successfully applied for the detection of Microcystin-LR in tap, pond, and river water samples.

**Table 1:** MOF-based coatings used for the SPME extraction of environmental samples.

Analyte	Material	Deposition Method	Matrix	Extraction Mode	Platform	LOD (ng/L)	EFs	References
BTEX	SOM-ZIF-8	physical adhesion	wastewater	HS	GC-FID	1.0–12	-	[48]
BTEX	_NS_ZIF-8^Si^	physical adhesion	river water	HS	GC-MS	0.02–0.21	-	[49]
BTEX	MIL-101-NH_2-_derived urchin-like nanoporous carbon	physical adhesion	pond water and river water	HS	GC-MS	0.08–0.36	-	[50]
BTEX, styrene, and trimethylbenzene	PAN/MIL-53(Al)@MOF@SBA-15/4,4′-bipyridine hybrid nanocomposite	in situ electrodeposition	tap water, mineral water, well water, and wastewater	HS	GC-FID	2.3–3.6	318–385	[51]
BTEX + 14 VOCs	MOF-199	in situ growth	air	DI	GC-MS	0.03–0.09 ^a^	-	[52]
16 PAHs and 11 nitro-PAHs	ZIF-8	sol–gel deposition	tap water, surface water, and wastewater	DI	GC-MS	0.3–27.0	-	[54]
3 PAHs and 5 PPCPs	ZIF-8	CVD deposition and in situ growth	wastewater	DI	GC-FID	600–2000	-	[55]
8 PAHs	ZIF-8	in situ electrodeposition	lake water	HS	GC-FID	10–54	-	[56]
6 PAHs	PANI/ZnO nanorods/ZIF-8	in situ growth	sewage water	EE-SPME/DI-SPME	GC-FID	8.2–134	-	[57]
6 PAHs	Co@ZIF-67	in situ growth and electrodeposition	snow, lake water, river water, and wastewater	DI	HPLC-UV	5–42	-	[58]
7 PAHs	MAF-66	physical adhesion	lake water and food	HS	GC-FID	0.1–7.5	127–3108	[59]
16 PAHs	MAF-5 and MAF-6	physical adhesion	wastewater and milk products	DI	HPLC-FLD	6–540	-	[60]
8 PAHs	HKUST-1 membrane	in situ growth and physical adhesion	lake water	HS	GC-MS	0.1–9.9	-	[61]
16 PAHs	PUM-210	physical adhesion	contaminated water	DI	GC-MS	0.50–3.7	300–14,950	[62]
10 PAHs	PI(Cu-DAT)	in situ growth and dip-coating	river water and fish muscle	HS/DI	GC-MS	0.3–2.1, 4.0–18.9	-	[63]
diazinon and chloropyrifos	PAN/Ni-MOF	post-synthetic electrodeposition	river water, farm water, groundwater, and beverages	HS	CD-IMS	200–300	-	[65]
ethion	ZIF-67 film	in situ electrodeposition	underground water and agricultural wastewaters	DI	SESI-IMS	100	-	[66]
6 OCPs	NU-1000	physical adhesion	river water and seawater	DI	GC-MS	0.011–0.058	972–2275	[67]
8 chlorobenzenes	Ni@NiO/PCs	physical adhesion	tap water and river water	DI	GC-MS	0.07–0.165	-	[68]
PFOA	ZIF-8, UiO-66, MIL88-A, and Tb_2_(BDC)_3_	in situ growth	contaminated tap water, rainwater, and seawater	DI	Direct-MS	11	-	[70]
8 PFASs	NH_2_-ZIF-8	physical adhesion	river water, seawater, and wastewater	DI	HPLC-MS/MS	0.15–0.75	-	[71]
11 PFASs	NH_2_-UiO-66(Zr)-hp	physical adhesion	tap water, river water, and pond water	DI	HPLC-MS/MS	0.035–0.616	6.5–48	[72]
9 PFASs	MIL-101-DETA-F	physical adhesion	tap water, river water, and wastewater	DI	UHPLC-MS/MS	0.004–0.12	70–112	[73]
Ibuprofen and diclofenac	Zr-MOF@GO	physical adhesion	river water	DI	GC-FID	1–30	-	[75]
3 PPCPs	Polyfam/Co-MOF-74 composite nanofibers	post-synthetic electrodeposition	wastewater and biological fluids	Thin film-SPME	HPLC-UV	30–200	24–37	[76]
3 PPCPs	MIL-101	physical adhesion	municipal wastewater	HS	GC-MS	4–60	-	[77]
6 methylsiloxanes and 7 musk fragrances	CIM-80(Al)	in situ growth	wastewater and seawater	HS	GC-MS	100–3500 ^b^	-	[78]
5 UV filters	NH_2_-UiO-66(Zr)	physical adhesion	river water and pond water	HS	GC-MS	0.2–2.1	865–3321	[79]
5 fluoroquinolones	ZIF-8	in situ deposition	tap water, river water, and wastewater	IT	HPLC-FLD	0.14–0.61	255–296	[80]
7 PCBs	ZIF-67 derived N-CNTCs	physical adhesion	river water	DI	GC-MS	0.10–0.22	-	[83]
5 PCBs	ZIF-8 derived HCNBs	physical adhesion	river water, pond water, and rainwater	HS	GC-MS	0.0017–0.0042	-	[84]
1-naphthol and 2-naphthol	HZ-PMOF	physical adhesion	urban water samples	HS	GC-MS/MS	1.0	-	[85]
methyl tert-butyl ether	PPy@MIL-101(Cr)	post-synthetic electrodeposition	soil	HS	GC-FID	0.01 ^c^	-	[86]
5 odorous organic compounds	MOF-74-C	in situ growth	tap water, lake water, and wastewater	DI	GC-MS	0.01–100	520–3000	[88]
6 substituted phenolic compounds	NH_2_-UiO-66(Zr) derived Zr/N-OMC	physical adhesion	river water and pond water	HS	GC-MS	0.21–1.7	-	[89]
8 PAEs	IL/UiO-66-NH_2_	in situ growth	river water, lake water, and bottled water	DI	GC-MS	0.2–0.4	-	[90]
Microcystin-LR	PAN/UiO@UiO_2_-N_3_-aptamer	post-synthetic electrodeposition	tap water, pond water, and river water	DI	LC-MS	3	-	[91]

^a^ μg/m^3^; ^b^ LOQ; LOD not provided; ^c^ ng/g.

## 4. Covalent Organic Frameworks (COFs)

In recent years, COFs [92] have emerged as a novel type of ordered crystalline and tunable porous materials: unlike MOFs, they consist of light elements (H, B, C, N, and O) connected by covalent bonds in which the pores are obtained by linking different groups in a cyclic manner. Although the number of developed MOF structures is higher compared to the number of developed COFs, one the major advantage in the use of COFs relies on the absence of metallic elements in their structure, thus making them more stable when exposed to water or solvents. In addition, the lack of a metallic center increases the surface/weight ratio and the greenness of the materials. Moreover, COFs can form either 2D or 3D constructs, whereas MOFs usually present a 3D architecture. Owing to their unique features in terms of thermal and chemical stability, high specific surface area, permanent porosity, and tunable frameworks, COFs are considered as promising coatings for the SPME extraction of different classes of compounds from highly complex matrices [30,32,93]. 

### 4.1. Synthetic Procedures

Similarly to MOFs, the morphology and properties of COFs strongly depend on both the organic building blocks and synthetic conditions, including reaction medium, time, temperature, and type of catalyst [30,32,93,94]. According to the dimensions of the building blocks, they can be classified into 2D structures and 3D consolidated networks. Two-dimensional COFs are based on rigid conjugated planar macrocyclic molecules, such as porphyrin, thiophene, phthalocyanine, and tetrazolium, presenting strong interlayer interactions, mainly π-π stacking. Both 2D and 3D COFs include different active functional groups, namely, B-O, C=N, C–N, and C=C [30,32,93]. They can be synthesized via boron or nitrogen condensation, triazine-based trimerization, metal-catalyzed coupling reactions, and Schiff base reactions between organic monomers.

Boron-based COFs consist mainly of boronated ester COFs, obtained through covalent interaction between catechol and polyfunctional boric acid. The main features of these materials are low density, high specific surface, and multiple binding sites; however, they suffer from low chemical stability, being that these COFs are usually unstable in humid environments and in air [12,93,95]. COFs based on Schiff base linkages encompass different organic ligands with substituents on the amine moiety, including imines, hydrazones, azines, and β-ketoenamines. Among them, imine-based COFs are based on the formation of C=N linkage though the condensation reaction between amino groups and aldehydes in the presence of a Lewis acid catalyst. These COFs are stable both in common organic solvents and water, providing superior stability compared to boron-based COFs. Imide-based COFs are based on the condensation between an amine and an anhydride at high temperatures. Amine-type COFs are less stable and require a laborious synthesis, which largely limits their application. Recently, novel β-ketoenamine-linked COFs have emerged, featuring high chemical and thermal stability owing to the presence of irreversible bonds, which cannot be hydrolyzed in water, acidic or basic environments [96,97]. Another important class is the triazine-based COFs, which are characterized by surface areas up to 2390 m^2^/g and high thermal and chemical stability. Finally, C=C linkage-based COFs have been developed, exploiting less reversible or irreversible poly-condensations processes: 2D sp^2^ C=C COFs have been obtained by condensation of aldehydes and benzyl cyanides in the presence of a base catalyst [32,93].

Up to date, different synthetic strategies have been proposed to produce COF-based SPME coatings. The most applied technique is solvothermal synthesis, due to the use of high-pressure conditions to promote the formation of the crystalline framework. Additional approaches have been suggested, among which microwave-assisted and sonochemical syntheses are gaining increasing interest, reducing the reaction time and preserving the porosity of the framework. Ionothermal synthesis has been also proposed for the preparation of triazine-based COFs: despite the green and solventless approach, this strategy requires very harsh reaction conditions and has limited applications due to the poor control of the crystallinity [32]. Finally, mechanochemical (MC) synthesis is a new mild, simple, and rapid approach to synthetize COFs by grinding reaction: high yields, great thermal stability, and promising crystallinity are the key features of this synthetic strategy, although limitations in terms of porosity and superficial area have been demonstrated [20,32,93].

### 4.2. COF Features for SPME Extraction

Among the interesting features of COFs, their low density, large surface area, and adjustable pore size and structure, as well as customizable properties, are the most attractive features that make them exceptional SPME materials. As previously reported, the thermal and chemical stability of COFs depend on their crystalline structure, particularly on both the bond strength and the electrostatic forces between the interlayers. Different methodologies have been proposed to enhance the stability of COFs, including the application of highly planar building blocks able to increase the interlayer force, the linkage conversion, and the conversion from reversible (e.g., imide-linked) to irreversible (e.g., amide-linked) COFs [14,30,95].

The most common typologies of COFs are 2D and 3D COFs. The 2D COFs exhibit intralayer covalent bonds and interlayer non-covalent interactions, such as π-π stacking. Adsorption usually occurs via hydrophobic interaction with planar aromatic analytes between the layers. In contrast, 3D COFs present a framework linked with strong covalent bonds and accessible open binding sites with a high surface area characterized by a hierarchical intrinsic nanoporosity. This feature increases the extraction selectivity and enrichment performance of the SPME coating compared to 2D frameworks due to the requirements in terms of steric complementarity [98,99]. Recent studies have introduced heteroporous COFs as an alternative solution for the simultaneous extraction of molecules with different sizes, thus enhancing both the selectivity and the extraction performance of the coating material. 

Pore dimensions can be tuned by changing the reaction conditions or the utilized building blocks; however, it has to be considered that micropores and small mesopores generally limit mass transfer and accessibility to the inner surface of the framework. Post-functionalization of the binding sites can also be performed to induce the formation of specific analyte–COFs interactions, improving both the extraction performance and selectivity [95]. This strategy can involve either the modification of the organic groups already present in the original framework or the incorporation inside the COF of predesigned structures able to host target analytes [20,95]. Finally, the use of a protective, thin layer mostly made by a polyimide polymer proved to be useful in improving both the mechanical strength and life span of COF-based coatings [100].

### 4.3. COFs as SPME Coatings for Environmental Applications

COFs as SPME coatings, prepared by physical adhesion, the sol–gel method, in situ growth, and chemical cross-linking, have recently attracted considerable attention for the analysis of organic pollutants, including polycyclic aromatic hydrocarbons [94,101,102,103,104,105,106,107,108,109,110,111,112], phenols [97,113,114,115], polybrominated diphenyl ethers [98,100,116,117,118,119,120], polyhalogenated biphenyls [99,121,122], pesticides [123,124,125,126,127,128], and PFASs [129,130,131] in environmental samples. The COF-based coatings used for the extraction of targeted compounds in environmental samples are summarized in Table 2.

#### 4.3.1. Extraction of Polycyclic Aromatic Hydrocarbons and Nitroaromatic Compounds

COFs have been proposed as novel SPME coatings for the extraction of PAHs from water and soil samples, based on either HS or DI modes. Recent studies investigated the HS extraction performances of different COF coatings toward low-molecular-weight PAHs [94,101,105,106,107]. Huo and coworkers developed a hydrazone-based covalent organic framework (BTCH-PTA-COF) coating by the sol–gel technique [101]. The adsorption of PAHs within the framework was mainly attributed to the π-π and hydrophobic interactions between the target compounds and the benzene rings and C=N bonds present within the COF. The material was characterized by a high surface area (1460 m^2^/g), mesoporous structure, and high hydrophobicity. Under the optimal extraction conditions, the BTCH-PTA-COF-coated fiber demonstrated a high extraction efficiency for PAHs in a water sample, with EFs in the 767–1411 range, LOQs in the 0.10–0.15 μg/L range, RSDs always lower than 9.6%, and recovery rates between 88.5 and104.8%, allowing for the sensitive analysis of target pollutants in river water samples. A facile and layer-by-layer self-assembly was proposed by Tian et al. to develop under very mild conditions a thin SPME film based on imine COFs (TFPA-TAPP-COF) [105]. The TFPA-TAPP-COF coating fiber consisted of tetra(4-aminophenyl) porphyrin and tris (4-formyl phenyl) amine: the use of very mild synthetic conditions together with good mechanical strength, remarkable thermal stability (up to 260 °C), and high extraction efficiency towards PAHs were the main features of the proposed material. The synthetic procedure allowed a close control over both the thickness of the coating and the number of assembled layers. The HS-SPME extraction performance of the novel COF material was assessed by extracting five model PAHs from aqueous samples, achieving LODs in the 6–24 ng/L range and RSDs < 10%. Finally, the HS-GC-FID method was applied for the analysis of spiked river water samples, obtaining recoveries in the 80.3–102.6% range. Another porphyrin-based COF was synthesized through a Schiff base reaction by Yu and coworkers [106] and deposited onto SS wires using a silicone sealant. An average narrow pore size of 8.7 nm was achieved, leading to an improvement in the selectivity of the material. PAH extraction was mostly related to π-π stacking, van der Waals interactions, and weak hydrogen bond formation between the amino group of the COF and the hydrogen atoms in the PAHs. The developed HS-SPME method showed LOQs in the range of 0.5–10 μg/L and good inter-day and fiber-to-fiber repeatability, with RSDs < 9%. Finally, the HS-SPME-GC-FID method was applied for the extraction of five model low-molecular-weight PAHs from both lake water and soil samples, obtaining recoveries in the 68–99% and 41–105% ranges, respectively. Recently, Yang et al. developed a COF based on 1,3,5-tris(4-aminophenyl)benzene and trimesoyl chloride (TAPB-TMC-COF) by amide coupling at room temperature. The coating was obtained the by physical deposition of the material onto etched SS wires and used for the HS-SPME-GC-MS determination of low-molecular-weight PAHs in water samples [107]. Analyte adsorption was mostly due to π-π stacking, with EFs in the 819–2420 range. The developed method exhibited good sensitivity (LODs in the 0.29–0.94 ng/L range) and linearity over three orders of magnitude. The HS-SPME-GC-MS method was applied for the analysis of PAHs from spiked river, pond, and wastewater samples, obtaining RSDs < 8% and recoveries in the 79–105% range, although a significant matrix effect was demonstrated for the wastewater samples. An imine-based COF-SCU1 material was prepared through a one-step synthesis and packed into a stainless-steel needle to be used as a needle trap device (NTD) for the extraction of PAHs from soil samples [94]. Compared to classical fiber extraction, the NTD proved to be a more robust microextraction tool, characterized by a higher sorption capacity and extraction efficiency. The validation of the COF-packed NTD-GC-FID method allowed for the achievement of LODs in the 0.01–0.05 ng/g range, excellent linearity over five orders of magnitude, and RSDs always lower than 13%. Finally, a ΣPAH concentration in the 0.86–2.3 μg/g range was obtained when soil samples collected from the neighboring ground of gas stations were analyzed.

**Figure 7:**
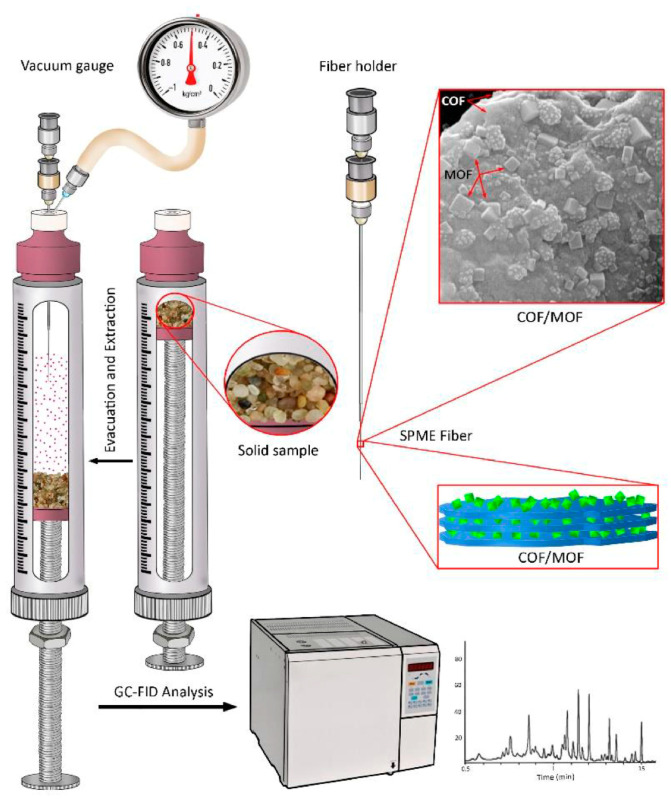
Schematic representation of ISV-HS-SPME extraction using hybrid MOF/COF coating, reprinted from [110].

Another interesting solution for the extraction of PAHs was based on the development of hybrid MOF/COF materials, which combine the outstanding features of MOF and COF structures, integrating the rigidity and high surface area of MOFs with the high flexibility and tunability of the COF surface. Different MOF/COF SPME coatings were obtained for the HS-SPME-GC-FID determination of PAHs from contaminated soil samples [108,109,110]. Koonani and coworkers developed a Zn-MOF/COF coating by solvothermal synthesis of the MOF precursor, followed by a further functionalization using ethylenediamine and a hydrothermal treatment with melamine and 1,4-benzenedicarboxylate to prepare the new MOF/COF hybrid material [108]. A similar approach was followed by Nouriasl at al. to obtain a porous Cu-MOF/COF coating using an amino-functionalized Cu-MOF as the starting material [109]. The coatings were obtained by the physical deposition of the material onto SS fibers using an epoxy glue. These synthetic strategies resulted in the COF assembly within the pores of the amino-MOF by in situ condensation using the amino groups present on the MOF surface as linkers to obtain stable covalent bonds between the MOF and the COF. The hybrid MOF/COF coatings showed higher extraction efficiency than both commercial fibers and individual materials, being able to interact with PAHs via π-π, van der Waals, and hydrophobic interactions. In addition, the presence of nitrogen atoms in the framework was exploited to promote electron donor–acceptor interactions with π-donor aromatic compounds, whereas the Zn or Cu metal sites could interact with the Lewis bases of the condensed aromatic rings. The HS-SPME-GC-FID method proposed by Koonani et al. was validated for the analysis of six PAHs, obtaining LODs below 1 ng/g and RSDs < 12%. The method was then applied for the analysis of contaminated soil samples, detecting the analytes up to 70 ng/g [108]. Compared to the previous study, the HS-SPME-GC-FID method proposed by Nouriasl et al. was characterized by lower LODs (in the 0.1–0.5 ng/g range), and higher precision, with RSDs below 8%, allowing for the determination of the investigated analytes in environmental samples at concentration levels up to 839 ng/g [109]. A hybrid COF/MOF (2DTP/MIL-101-Cr) coating was developed by Maleki et al. to be used for the extraction of BTEX and six model PAHs [110]. The MIL-101 MOF was obtained by hydrothermal synthesis, whereas the 2D COF was synthesized through metal-assisted and solvent-mediated synthesis. The hybrid material was obtained by ultrasonically mixing the two components, resulting in the embedding of the MOF within the COF framework. The material was physically deposited onto an SS needle to be used as an SPME coating for the in-syringe, vacuum-assisted HS-SPME (IS-VA-HS-SPME). The VA-HS-SPME relies on the application of a reduced pressure to improve the transfer rate of high-boiling-point components from the sample matrix to the headspace, effectively increasing the extraction efficiency of semi-volatile compounds (Figure 7). The hybrid material exhibited higher extraction performances than both the individual materials and commercially available PDMS and CAR/PDMS fibers. The optimized VA-HS-SPME-GC-FID method was validated, obtaining LOQs in the 7.1–17 ng/g and 0.23–3.7 ng/g ranges for BTEX and PAHs, respectively, with RSDs below 16%. When applied to the analysis of contaminated soil samples, both BTEX and PAHs were quantified at sub-trace levels, in the 0.26–3.6 μg/g and 0.02–1.8 ng/g ranges, respectively.

As for the DI-SPME extraction of PAHs from aqueous samples, novel COF-based coatings have been proposed [102,103,104,111,112]. Li and coworkers synthesized by ultrasonic-assisted reaction a COF based on 1,3,5-triformylphloroglucinol (Tp) and benzidine (BD), which was grafted on etched SS fibers using APTES [111]. The electroetching procedure resulted in an array of nanopores on the SS surface, promoting the grafting of the COF in the microwells. The SPME fibers were tested for the extraction of seven model PAHs from tap and lake water samples followed by GC-FID analysis. Validation resulted in LODs in the 1–5 μg/L range, RSDs < 10%, and recoveries in the 88–163% range. Feng et al. developed a triazine-based COF under solvothermal conditions, subsequently deposited onto SS wires using an epoxy resin. Finally, the SS wires were inserted in a peek tube to be used for the IT-SPME-LC-UV determination of different contaminants in environmental water samples [112]. The good extraction capability towards eight model PAHs, four estrogens, and eight plasticizers was assessed by achieving EFs in the 542–942, 190–929, and 68–1235 ranges, respectively. After optimization of the extraction conditions, the IT-SPME-LC-UV method was validated for the analysis of PAHs, obtaining LOQs in the 13–33 ng/L range, a fiber-to fiber reproducibility with RSDs ≤ 14%, and a wide linear range. The method was then applied for the analysis of tap, river, and rainwater samples; however, no analyte could be detected in unspiked samples. In another study, COF TpPa-1 was used as a template framework to develop a novel carbonaceous material [102]: the derived N-doped porous carbons (TpPa-1–1000) were deposited onto SS wires by physical deposition and used for the extraction of PAHs from soil samples. The TpPa-1–1000 material featured a high nitrogen content (5.62% atomic), a specific surface area of 435.6 m^2^/g, a uniform pore size distribution (3.9 nm), and a porous layered structure, thus increasing the number of accessible binding sites for promoting the interaction of the adsorbent with the target analytes. Compared to commercially available materials, the coating provided enhanced EFs (in the 1686–41,294 range) due to both the high degree of graphitization and the formation of electron-donor–acceptor interactions. The validation of the DI-SPME-GC-MS method resulted in LOQs in the range of 10.4–28.5 μg/kg of soil sample, and RSDs lower than 12%. A DI-SPME-GC-MS method was also proposed by Zang and coworkers, who tested three different COF-modified graphitic carbon nitride (g-C_3_N_4_) materials for the extraction of PAHs from aqueous samples [103]. The g-C_3_N_4_@TpBD coating provided the best extraction performances toward the investigated PAHs. The developed coating was characterized by higher extraction performances than commercial 85 μm PA, 60 μm PDMS/DVB, and 100 μm PDMS fibers, probably due to the π-conjugated structure of g-C_3_N_4_, allowing for π-π stacking and hydrophobic and electrostatic interaction with the target analytes. The DI-SPME-GC-MS method showed LOQs in the 0.07–0.17 μg/L range, good linearity (over a three orders of magnitude range), and RSDs always lower than 11%. Finally, the method was applied for the analysis of river, lake, well, and rainwater samples and melted snow, allowing the detection of naphthalene and acenaphthene at the sub-μg/L level.

A multi-component coating was developed by Sun and coworkers: carbon fibers were coated with titania nanorod arrays (NARs) and functionalized with either biochar nanospheres or COF nanospheres [104]. The obtained hybrid fibers were introduced in a PEEK tube to be used for the IT-SPME extraction of PAHs before LC-UV analysis. The functionalization with either the biochar or COF nanospheres proved to be useful in increasing the BET surface from 10.07 m^2^/g to 15.97 m^2^/g and 32.75 m^2^/g, respectively. The extraction capability of the different materials was tested for the extraction of PAHs, estrogens, and bisphenols, resulting in the selection of TiO_2_NARs–CFs, biochar nanosphere– TiO_2_NARs–CFs, and COF nanosphere–TiO_2_NARs–CFs as the best coatings for the extraction of the different classes of compounds. The IT-SPME-LC-UV method was validated obtaining LODs in the 5–10, 1–5, and 5–100 ng/L ranges for PAHs, estrogens, and bisphenols, respectively, and RSDs always lower than 15%.

Hydrogen-bonded organic frameworks (HOFs) are other emerging SPME coatings consisting of organic or metal–organic architectural units connected by hydrogen bonds. They are gradually developing into an attractive new type of porous crystalline material for the determination of nitroaromatic compounds. To increase the extraction selectivity toward this class of analytes, Hu and coworkers developed an SPME coating based on the self-assembly of melamine and 1,3,6,8-tetra(4-carboxylphenyl) pyrene to obtain a MA/PFC-1 HOF [132]. The use of melamine promoted the formation of hierarchical micropores with dimensions in the 5–20.0 Å range, a surface area of 1550(±26) m^2^/g, and strong hydrogen bonds between the constituting units. The HOF coating significantly improved the extraction performance of five model nitroaromatic compounds via polar, electrostatic, hydrogen bonding, π–π stacking, and C–H⋅⋅⋅π interactions between the analytes and the adsorbent material. The HS-SPME-GC-MS method was validated, obtaining LOQs in the 4.3–20.8 ng/L range and RSDs ≤ 9%, allowing for the detection of *p*-nitrochlorobenzene and *p*-nitrotoluene and p-nitrochlorobenzene in water samples at the sub-μg/L level.

#### 4.3.2. Extraction of Phenols and Derivatives

Phenols, phenolic compounds, and synthetic derivatives are characterized by very different chemical and physical properties. The main challenge in their detection is related to their hydrophilicity and low concentrations in environmental matrices. In this context, COFs as SPME coatings could be promising materials to address both enrichment and selectivity issues [97,113,114,115].

Recent studies investigated the extraction capabilities of two different COFs, namely a spherical TPB-DMTP-COF [113] and a piperazine-linked, copper-doped phthalocyanine metal covalent organic framework (CuPc-MCOF) [114], to be used as SPME coatings for the extraction of phenols and chlorophenols (CPhs). The spherical TPB-DMTP-COF was synthesized in an organic solvent at room temperature and deposited onto etched SS wires by means of a silicone sealant. A uniform spherical superstructure (1–2 μm) with a strong π-π stacking architecture between the layers, a pore size of 2.54 nm, and a BET surface of 1560 m^2^/g, higher than that of solvothermal synthesized COFs, were the main features of the developed coating [113]. The SPME fibers were applied for the HS extraction of phenols and CPhs, obtaining EFs in the 1741–4265 range. The adsorption was mostly driven by hydrophobicity and steric hindrance effects, with the formation of π–π, van der Waals interactions, and hydrogen bonds between the analytes and the material. Validation demonstrated LOQs and recoveries in the 0.016–0.050 ng/L and in the 81–116% ranges, respectively, with RSDs ≤ 10%. When the HS-SPME-GC-MS/MS method was applied for the analysis of environmental samples, no analyte was detected in drinking and reservoir water, whereas underground water samples were contaminated by CPhs (in the 0.6–22.7 ng/L range). Wang and coworkers synthesized a CuPc-MCOF coating via Buchwald–Hartwig cross-coupling and deposited it onto etched SS fibers using a polyimide resin. The coating was used for the EE-SPME of CPhS from water and biological samples, namely oysters and prawns [114]. The high hydrophobicity of the COF-based material was ascribed to its conjugated structure and abundant fluorine atoms, whereas the presence N and of Cu atoms within the framework provided free metal sites for coordination binding. During EE-SPME, the analytes underwent mass transfer, thus increasing the enrichment of chlorophenols onto the fiber coating, acting as an electrode. The extraction performance of the developed coating was better than that of commercially available SPME fibers, with EFs in the 339–988 range. The EE-SPME-GC-MS/MS method was validated, obtaining LOQs at low ng/L levels, linearity over three orders of magnitude, and good precision (RSD < 9%). Finally, the method was applied for the analysis of seawater samples, detecting trichlorophenols in a 6.8–30.1 ng/L range with a recovery rate ranging from 90% to 113%. Similarly, when oysters and prawns were analyzed, 2,4-dichlorophenol, 2,4,5-trichlorophenol, 2,4,6-trichlorophenol, and 2,4,5,6-tetrachlorophenol were detected at low ng/L levels.

Guo and coworkers developed a TpBD COF based on an MC approach and the coating was obtained either by in situ growth or physical deposition onto SS wires [115]. Depending on the deposition procedure, different morphologies were obtained: in situ deposition resulted in a highly porous structure with a uniform section, whereas a less uniform coating with a smooth cross-section characterized by a less porous structure was observed for physical deposition, probably due to pore obstruction by the silicone adhesive. Both coatings were tested for the HS-SPME-GC-MS analysis of 2-nitrophenol, 2,6-dimethylphenol, 2,4-dimethylphenol, 2,4-dichlorophenol, and 2,4,6-trichlorophenol. The extraction performance of the in situ grown coating was superior to both the physically deposited COF and 85 μm PA commercially available fibers, mainly due to the formation of π–π interactions and hydrogen bonding. The reliability of the method for the determination of the investigated analytes was attested at trace level, obtaining LOQs in the 1.3–2.4 ng/L range with RSDs < 8%. The feasibility of the method was further demonstrated by analyzing water and soil certified reference materials, obtaining phenol concentrations in compliance with the reference value.

Finally, Gao and coworkers developed a COF–graphene oxide composite material (COF-GO) deposited onto a glass fiber using a silicone sealant to be used as an SPME coating for the analysis of bisphenol A [97], a well-recognized endocrine-disrupting compound used in the polymer and packaging industries. The hybrid material was synthesized by a three-step synthesis involving: (i) the preparation of the amino-functionalized GO using APTES, (ii) the addition of Tp between the layers of the material, and (iii) the in situ growth of the COF. The functionalization of GO with the COF increased the BET surface, pore volume, and the interlayer spacing from 353 m^2^/g, 0.16 g/cm^3^ and 8.4 Å to 824 m^2^/g, 0.46 g/cm^3^, and 14.7 Å, respectively, thus providing more active sites and faster mass transfer processes. The obtained SPME fibers were used for the CFDI-MS determination of bisphenol A in water samples, obtaining an extraction performance 2.2–4.7 times higher than TpBD and GO and 2.1–4.9-fold higher than 65 μm PDMS/DVB-, 85 μm PA-, and 100 μm PDMS-coated fibers. The validation of the DI-SPME-CFDI-MS method resulted in LOD and LOQ values at sub-trace levels (22.2 and 73.9 ng/L, respectively) and RSDs < 6%. When applied to the analysis of river and seawater samples, bisphenol A was detected in all samples in the 0.1222(±0.0077)–4.794(±0.072) range, with recovery rates in the 95–107% range.

**Figure 8:**
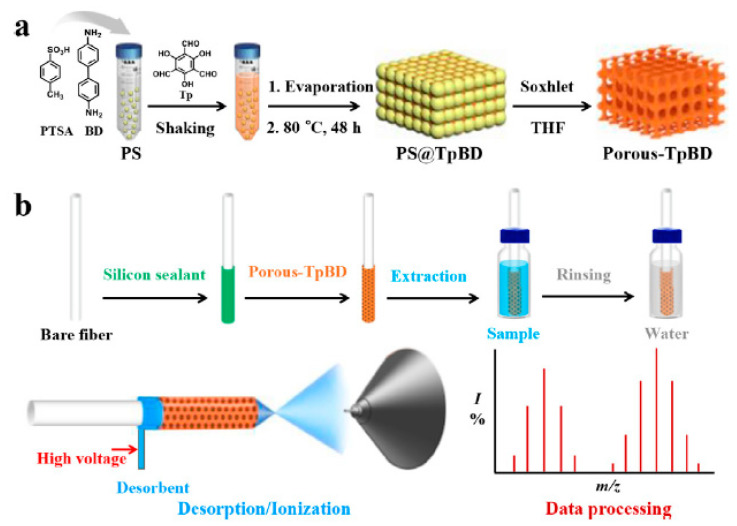
Schematic illustration of the (**a**) synthesis and (**b**) coating and SPME-CFDI-MS procedure for analysis of TBBPA analogs, reprinted with permission from [98].

The same research group developed different COF-based coatings for the analysis of tetrabromobisphenol A (TBBPA) and its analogs in environmental matrices using ambient mass spectrometry [98,119,120]. These pollutants have gained great attention due to their worldwide distribution, persistence, bioaccumulation, immunotoxicity, and neurotoxicity. TpBD-COF and multicomponent COFs were tested for the extraction of TBBPA and its analogs from environmental water samples. In an early study, a TpBD-COF was tested as an SPME coating for the DI-SPME–constant flow desorption ionization–mass spectrometry (CFDI-MS) analysis of TBBPA in water samples, obtaining LOD and LOQ values of 0.92 and 3.1 ng/L [119]. To improve the extraction performance of the SPME, different multi-component COFs containing Tp, Pa, and BD were tested for the analysis of TBBPA derivatives in water samples. The main feature of the multicomponent COF is the pore size tunability by changing the ratio of the organic linkers: the best performance was obtained using the TpPaBD_50_ framework, exhibiting extraction efficiencies up to four times higher than TpPa and TpBD COFs [120]. A further study demonstrated that the best analytical performance could be obtained using porous TpBD, synthesized using a polystyrene sphere (PS) template-assisted method (Figure 8) [98]. Porous TpBD was characterized by a hierarchical porous structure with a BET surface and pore volume higher than those of the conventional TpBD (797 m^2^/g and 0.75 cm^3^/g vs. 638 m^2^/g and 0.41 cm^3^/g). The extraction capabilities of the COF were strongly related to its hierarchical porous structure, which featured micro-, meso-, and macropores, promoting the diffusion/mass transfer processes. The CFDI-MS-based method for the analysis of four TBBPA analogs showed good performance, with LOQs in the 0.4–3.2 ng/L range and good precision, with RSDs < 8%. When applied to the analysis of environmental samples, contamination was assessed for all the river water samples, with a concentration up to 51 ng/L and, for two out of three seawater samples, concentration levels up to 66 ng/L.

#### 4.3.3. Extraction of Polybrominated Diphenyl Ethers and Polyhalogenated Biphenyls

Polybrominated diphenyl ethers (PBDEs) and polybrominated biphenyls (PBBs) have been widely used as flame retardants in manufacturing since the mid-1990s; now, their use is strictly regulated due to their toxicity, high persistence in the environment, and impact on ecosystems [133]. Owing to their potential ecotoxicological effects on organisms, sensitive methods based on gas chromatography-negative chemical ion mass spectrometry (GC-NCI-MS) and COF-based SPME coatings have been developed to increase both selectivity and extraction efficiency, allowing for the detection of these pollutants at trace levels.

**Figure 9:**
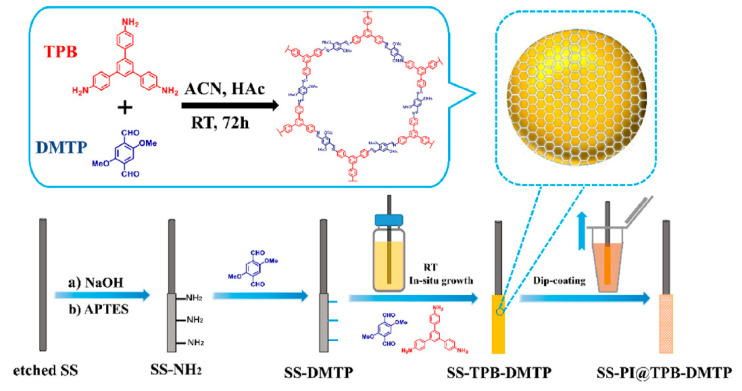
Schematic representation of the synthesis of the PI@TPB-DMTP coating, reprinted with permission from [100].

In this context, a TpPa-based coating was developed by Liu and coworkers [116] using a solvothermal reaction followed by physical adhesion on the fiber support by means of a silicone sealant. The material showed a flower-shaped morphology, high porosity, and a BET surface of 625 m^2^/g. The coating was tested for the DI-SPME-GC-NCI-MS analysis of five model PBDEs, allowing for the extraction of the analytes via hydrophobic, van del Waals, and π−π interactions, with EFs in the 2035(±22)–6859(±67) range. The selectivity of the coating was explained on the basis of the size complementarity between the pores of the material (approximately 1.25 nm) and the PBDEs (average size 0.81 nm). Validation of the optimized method resulted in LOQs in the 0.019–0.074 ng/L range and RSDs < 10%. The analysis of environmental water samples did not show the presence of the analytes in groundwater and drinking water, whereas concentration levels in the 0.50–2.18 ng/L range were reported in pond water. Song et al. developed a coating based on polyimide and a TAPB-DMTP COF (PI@TAPB-DMTP) supported on SS wires for PBDE extraction from water samples [100]. The etched SS fibers were pretreated with APTES to obtain an amino-functionalized surface to be used for the grafting of DMTP, followed by in situ growth of the COF at room temperature. A PI protective layer was finally deposited onto the fiber (Figure 9). The characterization of the coating revealed a very high BET surface (1880 m^2^/g) and the presence of mesopores with an average diameter of 2.2 nm, suitable for hosting the analytes. The PI@TPB-DMTP coating exhibited a high extraction performance with EFs in the 1470–3555 ng/L range, benefiting from strong hydrophobic effects, π−π stacking, enhanced surface area, and porosity. The optimized DI-SPME-GC-NCI-MS method was validated for the analysis of six PBDEs, obtaining LOQs in the low ng/l level, i.e., 0.027–0.063 ng/L, with RSDs lower than 9%. Despite the sub-trace detection limits, all the investigated PBDEs were below the LODs in the analyzed river, lake, and wastewater samples.

The use of poly(ionic liquid)s (PILs) to functionalize the surface of COFs was proposed by Su et al. [117]: two vinyl-decorated COF analogs (COF-β and COF-γ) were synthesized by solvothermal treatment, and then photo-initiated polymerization was performed to synthesize two series of poly(1-vinyl-3-methylimidazolium bis ((trifluoromethyl) sulfonyl) imide)-based hybrids (COF-PIL) with different mass ratios of IL/COF. Finally, the SPME coating was obtained by physical deposition using a silicone sealant. The use of co-polymerization to obtain covalently composited hybrids resulted in stable PIL@COF bonds. Interestingly, the COF-β and COF-γ hybrids showed different morphologies, namely, spherical and sheet stacking with a hierarchical porous structure, with PIL decorating the pores and interlayer volume. Despite the presence of PIL decreasing the BET surface area of the materials, the functionalization generally improved the extraction performance of the coating. This behavior was ascribed to the increased affinity between the COF surface and the adsorbed PBDEs, able to interact via weak hydrogen bonding, π-π stacking, and Van der Waals forces. The COF-γ-PIL exhibited a superior extraction capability compared to the COF-β-PIL: this behavior could be explained considering that the sheet-like stacking morphology featured shorter diffusion paths and broader pore size distribution, improving the accessibility of adsorption sites and mass transfer processes. The COF-γ-PIL-based coating also demonstrated a higher extraction capability when compared to commercially available coatings, namely, 100 μm PDMS, 65 μm PDMS/DVB, and 75 μm CAR/PDMS fibers, providing EFs in the 913–3625 range. Finally, the DI-SPME-GC-MS method was validated, obtaining LOQs in the 0.0070–0.046 ng/L range and excellent precision (RSDs < 8%), and successfully applied for the analysis of river, lake, and seawater samples.

Zhou and coworkers developed a coating based on TAPB—2,5-dimethoxyterephaldehyde (DMTP) COF using 2,5-dimethoxybenzaldehyde (DBA) as a modulator for the extraction of PBBs [118]. The material was fabricated at room temperature and deposited onto etched SS wires by means of a silicone sealant. The use of a modulator allowed the optimization of the COF microstructure in terms of specific surface area (BET values in the 1545–1611 m^2^/g range, using different modulator percentages), surface hydrophobicity (contact angles higher than 90° with DBA higher than 40%), and morphology (in terms of particle size). The material containing 40% DBA showed EFs higher than those obtained using the unmodulated COF (in the 4400–11,360 range). Adsorption occurred through π-π interactions, hydrophobic effects, and electrostatic interactions between the material and the analytes. The optimized HS-SPME-GC-MS method was validated, achieving LOQs in the range of 0.12–0.94 ng/L, precision with RSDs < 8%, and recoveries in the 80–120% range, thus allowing the determination of 3-bromo-1,1′-biphenyl and 4,4′-dibromo-1,1′-biphenyl at concentration levels in the 0.33–1.1 and 2.6–3.0 ng/L ranges, respectively.

COF-based SPME coatings have also been developed for the selective extraction of PCBs [99,121,122]. Lv and coworkers developed a TpPa-functionalized PAN membrane (PAN-SiO_2_@TpPa) [121]: the COF was grown in situ on the PAN-SiO_2_ electrospun nanofiber membrane, which was deposited onto etched SS fibers using a resin glue. Functionalization provided the COFs with improved properties in terms of BET area (33 m^2^/g vs. 108 m^2^/g for PAN-SiO_2_ and TpPa@PAN-SiO_2_, respectively), average pore size (35.1 ± 5.1 vs. 9.8 ± 1.3 nm) and pore volume (0.009 vs. 0.104 cm^3^/g). The TpPa@PAN-SiO_2_ coating showed extraction capabilities up to three times higher than the unfunctionalized membrane for the six investigated PCBs. The adsorption was obtained by the synergistic effect of hydrophobic interaction, π-π stacking, and hydrogen bonding. Under optimized conditions, the HS-SPME-GC-ECD method exhibited LOQs in the 0.1–5 ng/L range, EFs in the 2714–3949 range, and precision with RSDs< 9%. No PCB could be detected in river, lake, and seawater samples, whereas the analysis of spiked matrices resulted in recoveries in the 73–126% range. Su et al. synthesized a chlorine-functionalized COF coating onto etched SS wires pretreated with APTES [122]: the COF was grown in situ using Tp, 2,5-dichloro-1,4-phenylenediamine, mesitylene, and 1,4-dioxane, obtaining a chlorinated analogue of TpPa-1. Using the chlorine-functionalized COF, EFs in the 699–4281 range were achieved, higher than those obtained using the TpPa-1 and commercially available polymeric coatings (100 μm PDMS and 50/30 μm DVB/CAR/PDMS fibers). This behavior could be ascribed to π-π stacking and hydrophobic interaction between the target molecules and the coating, presenting mesopores able to accommodate PCB molecules. The HS-SPME-GC-MS method exhibited excellent performance for the determination of 17 PCBs, with LOQs in the 0.005–0.029 ng/L range, a linearity over five orders of magnitude, and good precision, with RSDs ≤ 9%. Finally, the method was applied for the analysis of sea, river, and reservoir water, detecting PCB-209 in all samples at sub-ng/L concentration levels. Finally, Lu et al. developed an SPME coating using a 3D COF [99]: four different 3D COFs were synthesized using tetra (*p*-aminophenyl) methane (TAM) and aldehyde reagents (Tp, 1,3,5-triformylbenzene, terephthalaldehyde and 1,1’-biphenyl-4,4’-dicarbaldehyde) under solvothermal conditions and each coating was deposited onto etched SS fibers using an epoxy glue. The TpTAM coating showed a higher BET surface area (537.2 m^2^/g), larger pore volume (0.32 cm^3^/g), sharper pore size distribution (0.68 nm), and higher EFs (6940–10,305) compared to the other developed materials. The adsorption of both planar and non-planar PCBs was enhanced by π-π stacking, hydrophobic interactions, halogen bonds, and steric complementarity between host and guest. Finally, the selectivity of the material towards the investigated analytes was proved by the achievement of EFs lower than those calculated for PCBs and biphenyl when structural PCBs analogs, including *o*-dichlorobenzene, naphthalene, anthracene, and pyrene were considered. An excellent HS-SPME-GC-MS method performance was obtained, with LOQs in the 0.004–0.066 ng/L range, recoveries in the 85–117% and 85–115% ranges for water and soil, respectively, and precision, with RSDs < 11%.

#### 4.3.4. Extraction of Pesticides and Insecticides from Water Samples

The use of COFs as key materials for SPME devices has been proposed as a valid alternative to the MOF-based coatings (described in Section 3.3.3), and also for the selective extraction of OCPs and OPPs at trace levels in environmental matrices [123,124,125,126]. Xin and coworkers developed a ketoenamine COF (Tp−Azo−COF) to be used as an SPME coating for the extraction of OCPs [123]. The COF was obtained by solvothermal treatment and deposited onto etched SS wires using an epoxy resin. The adsorbent material showed a high percentage (5.8%) of electronegative N atoms, facilitating the formation of halogen bonds between the analytes and the coating, a BET area of 1218 m^2^/g, and high porosity, with a total pore volume of 0.689 m^3^/g. The Tp−Azo−COF-coated fiber exhibited higher EFs compared to commercial fibers, ranging from 1061 to 3693 for the five investigated OCPs. DI-SPME sampling using this fiber followed by GC-MS/MS analysis of OCPs resulted in LOQs in the 0.005–0.26 ng/L range and good precision (RSDs < 11%). Green tea, milk, tap, and well water samples were analyzed by the validated DI-SPME-GC-MS/MS method: no analyte was detected in real samples, whereas recoveries in the 83.4−101.6% range were obtained on spiked matrices. A novel porous COF coating synthesized by condensation between cyanuric chloride, 4,4′-ethylendianiline, and 3,4,9,10-perylenetetracarboxylic dianhydride was devised by Tabibi and Jafari [124]. The COF was deposited onto SS wires using a silicon glue solution. The DI-SPME extraction was coupled with GC-CD-IMS analysis. The coating was used for the extraction of trifluralin (a common herbicide) and chlorpyrifos from agricultural wastewater and vegetables. Validation resulted in LOQs of 0.45 and 0.50 μg/L, and EFs of 1950 and 2123 for trifluralin and chlorpyrifos, respectively. A good precision was calculated, with RSDs below 11%. Finally, chlorpyrifos was detected in agricultural wastewater (1.2 μg/L) and both analytes were quantified in cucumber (2.4 and 5.6 μg/L for trifluralin and chlorpyrifos, respectively). A magnetic COF nanohybrid (NiFe_2_O_4_@COF) was developed for the SPME extraction of triclosan and methyltriclosan in environmental water and human urine samples [125]. Magnetic NiFe_2_O_4_ particles were synthesized by hydrothermal synthesis and the COF was grown in situ under ambient conditions by aldehyde-/amine-based condensation using terephthaldicarboxaldehyde and 1,3,5-tris (4-aminophenyl) benzene. The NiFe_2_O_4_@COF hybrid nanocomposite was physically deposited onto fused silica fibers. The core-shell nanohybrid-based coating was characterized by a pore diameter of 3.9 nm and BET surface (169.7 m^2^/g) larger than those of a pristine COF (1.2 nm and 58.4 m^2^/g, respectively). The extraction selectivity of the NiFe_2_O_4_@COF coating was demonstrated by comparing the EFs of the target analytes with those of five pyrethroid pesticides: EFs of 279 and 334 were calculated for triclosan and methyltriclosan, respectively, whereas values in the 76–147 range were obtained for the other pyrethroids. The adsorption of the analytes was ascribed to hydrophobic and strong π-π interactions between the COF shell and phenyl groups of the OCPs, leading to a higher extraction performance compared to commercially available fibers. The DI-SPME-GC-ECD method exhibited LOQs of 23 and 3.3 ng/L for triclosan and methyltriclosan, respectively, allowing for the detection of the investigated analytes in tap, river, and barreled water samples at the sub-μg/L level. Yang and coworkers developed an ultrastable crystalline, quinoline-linked 2D COF (COF-CN) for the selective extraction of 14 OCPs from environmental water samples [126]. The COF was synthesized based on a cycloaddition approach using TPB and 2,5-dimethoxyterephthalaldehyde as building blocks, followed by post-synthetic functionalization using 4-ethynylbenzonitrile. The SPME coating was fabricated by a two-step deposition using a PDMS layer for the physical deposition of the COF. Although post-synthetic functionalization led to a reduction in both the BET surface and pore volume of the material (700 m^2^/g and 1.2 nm for the COF-CN vs. 1983 m^2^/g and 2.5 for the unfunctionalized COF), the COF-CN material showed higher thermal stability and increased hydrophobicity. The developed COF exhibited EFs higher than commercially available coatings: values in the 540–5065 range were calculated, benefitting from suitable pore size, π-π stacking, and a larger specific surface area. The HS-SPME-GC-MS/MS method was validated for 14 OCPs, obtaining LOQs in the 0.0030–45.13 ng/L range and good precision (RSDs < 12%). Finally, the validated method was applied for the determination of OCPs in water samples from various cities in Henan Province, detecting six OCPs in all samples, hexachlorobenzene and aldrin in all samples except one, and heptachlor and 4,4′-DDT in some urban samples. 

Trace detection of insecticides is another key issue for environmental monitoring due to their worldwide use, high stability, and high toxicity, resulting in a strong impact on the aquatic ecosystems [127,128]. Song et al. developed an SPME coating based on a trifluoromethyl-grafted COF named COF-(CF_3_)_2_ for the extraction of benzoylurea insecticides from water samples [127]. The COF scaffold was synthesized under solvothermal conditions using 2,5-Dibromoterephthaldehyde and 1,3,5-tris(4-aminophenyl) benzene, post-synthetically functionalized through the Suzuki–Miyaura cross-coupling reaction under mild condition and deposited onto SS wires using a silicone glue. Although the functionalization with the trifluoromethyl group decreased both the BET surface and pore size from 1991 m^2^/g to 1636 m^2^/g and from 2.5 nm to 2.0 nm, respectively, a strong increase in the hydrophobicity as well as in the chemical and thermal stability was observed. The COF-(CF_3_)_2_ coating showed higher EFs (in the 44–105 range) compared to unfunctionalized COF and up to three times higher than those calculated using commercially available fibers, due to strong hydrogen bonds and fluorine–fluorine interactions between the COF coating and the analytes. In addition, hydrophobic, π-π stacking interactions, C–H⋯π interactions, and C–F⋯π interactions could be established. The DI-SPME-UHPLC-MS/MS method was validated, obtaining LOQs in the 0.20–1.70 ng/L range, recoveries in the 76.2–107.6% range, and good precision (RSDs ≤ 11%). The method was applied for the analysis of wastewater, river, lake, pond, and farmland water: the insecticides were detected in all the samples except for river water, with concentrations up to 33.2 ng/L. Eleven pyrethroid insecticides were extracted by Han and coworkers using a sea-urchin-like COF (COF_TDBA-TTL_) [128]. The COF was synthesized under solvothermal conditions and deposited onto SS wires using a silicone adhesive. The coating interacted with the investigated pyrethroid insecticides through electrostatic, hydrophobic, and π-π interactions, and hydrogen bonding, showing extraction performance up to 446 times higher than commercially available coatings. The DI-SPME-GC-MS method was validated, obtaining LOQs in the 0.720–5.61 ng/L range, EFs in the 2584–7199 range, and RSDs ≤ 13%. Finally, tefluthrin, cyhalothrin, acrinathrin, permethrin, and etofenprox were detected in Pearl River water samples at a concentration of 7.54, 9.96, 7.70, 10.3, 17.2, and 11.0 ng/L, respectively.

#### 4.3.5. Extraction of per- and Polyfluorinated Alkyl Substances

The use of COFs as SPME coatings for the extraction of PFASs has been proposed as a valid alternative to the use of MOFs, featuring a highly hydrophobic and π electron-rich structure [129,130,131]. In this context, a novel dioxin-linked COF (TH-COF) was synthesized by microwave-promoted nucleophilic substitution of 2,3,5,6-tetrafluoro-4-pyridinecarbonitrile with 2,3,6,7,10,11- hexahydroxy triphenylene [129]. The COF was then deposited onto SS wires using an epoxy resin and the coating was applied for the DI-SPME-UHPLC-MS/MS analysis of perfluoroalkyl carboxylic acids (C_4_–C_10_), perfluorobutanesulfonic acid, and perfluorooctanesulfonic acid. The use of microwave conditions strongly increased the BET surface of the material (from 576 m^2^/g to 1254 m^2^/g), obtaining pores with a diameter of 2 nm and a reticulate morphology. PFASs were adsorbed via van der Waals forces and strong hydrogen bonding between the pyridine N atoms of TH-COF and the analytes. Method validation resulted in very low LOQs (0.0066–0.015 ng/L), recoveries in the 89.5–105% range, and good precision, with RSDs ≤ 8%, allowing for the ultra-trace analysis of pollutants in river, drinking, and underground water samples: perfluoropentanoic acid and perfluorobutanesulfonic acid were detected only in the river water samples at a concentration of 0.480 ng/L and 0.451 ng/L, respectively. Song and coworkers developed a multifunctionalized COF, featuring an amide group and perfluoroalkyl-chain-functionalized chains (COF-NH-CO-F9), to be used for the extraction of anionic, cationic, and zwitterionic PFASs [130]. The material was obtained under solvothermal conditions and deposited onto SS wires using silicone elastomer. A BET surface of 681 m^2^/g, an interlayer distance of 4.8 Å, and an average pore size of 24 Å were observed, allowing for a size complementarity with the target analytes. In addition, the functionalization with fluoroalkyl chains increased the hydrophobicity of the surface and was beneficial for the selective enrichment of PFASs, which interacted with the coating via electrostatic, hydrophobic, fluorine−fluorine, and π−CF interactions, as well as through hydrogen bonding between N−H···F and N−H···O groups. The EFs for the investigated compounds were in the 66−160 range, up to 57 times higher than commercially available coatings, the non-fluorinated COF analogue, and the COF-(CF_3_)_2_ coating described in Section 4.3.4. By coupling DI-SPME with UHPLC-MS/MS, LOQs in the 0.01−0.65 ng/L range, recovery rates in the 77.1–108% range, and RSDs ≤ 11% were obtained. The method was then applied for the analysis of tap, river, lake, pond, and farmland water, and wastewater samples, detecting total PFAS concentrations in the 14.5–96.3 ng/L range. A triazine-core-based, F-functionalized COF (COF-F-1) was synthesized by Hou and coworkers at room temperature and physically deposited onto an SS probe to be used for the nanoESI-MS analysis of PFASs [131]. After the COF deposition, a chitosan polymeric layer was applied, enhancing the hydrophilicity, biocompatibility, and stability of the SPME probe. The COF-F-1 presented hydrophobic channels, fluorine-rich subunits, and electron-deficient groups containing nitrogen, able to interact with PFASs via fluorine−fluorine and electrostatic interactions, as well as hydrogen bonding. A good complementarity between the pore dimensions of the coating (2.8 nm) and the molecular sizes of the investigated C_7_−C_18_ PFASs (9.4–23.8 Å) was observed. The nanoESI-MS method was applied for the analysis of C_7_–C_10_ perfliuoroalkylsulfonic acids and C_7_–C_18_ perfluoroalkyl carboxylic acids, obtaining EFs in the 105−4538 range, indicating a high enrichment capacity of the developed coating towards PFAS. The highest values obtained for the perfluoroalkylsulfonic acids were ascribed to the hydrogen bonding between the oxygen atoms of the guest molecules and the protonated nitrogens within the COF. LOQs in the 0.06−3 ng/L range were obtained, together with recoveries in the 84−113% range, and RSDs below 12%, allowing for the detection of C_8_–C_18_ PFASs in the environmental sample with concentrations in the 5.4–38.6 ng/L range.

#### 4.3.6. Extraction of Other Compound Classes by COF-Based SPME

Phthalic acid esters (PAEs) are a class of plasticizers widely used in the polymer industry. Different compounds have been included in the list of priority substances by the US EPA due to the potential endocrine-disrupting activity; therefore, the development of new analytical methods able to detect these compounds at trace levels in environmental and biological matrices is demanded [134,135]. To improve their enrichment and selective extraction, Yu and coworkers developed different β-ketoenamine-linked COF coatings [96]. Four different COFs were tested, all synthesized by MC to obtain a clay-like material that was deposited onto APTES-pretreated SS fibers, obtaining covalently bonded COF coatings. The best performance was obtained using Tp and 4,4′-diamino-p-terphenyl (TpTph-COF). The material showed high hydrophobicity, a BET surface of 267.6 m^2^/g, and an average pore diameter of 2.52 nm. The analytes could be adsorbed via hydrogen bonding, π-π and hydrophobic interactions, together with a size-matching effect to guarantee a good extraction selectivity. The DI-SPME-GC-MS/MS method was validated, obtaining LOQs in the 0.2–0.5 ng/L range, EFs in the 1140–3720 range, and RSDs always lower than 10%. The analysis of environmental samples resulted in the detection of dipentyl phthalate (0.27 ng/L) and butyl benzyl phthalate (11.62 ng/L), diisohexyl phthalate (0.33 ng/L), dipentyl phthalate (1.65 ng/L), and butyl benzyl phthalate (2.34 ng/L) in lake water. 

Porphyrin-based COFs were proposed as novel materials to be used for the EE-SPME extraction of PAEs [136]: the coating was obtained by in situ electropolymerization onto etched SS fibers, obtaining a fine tuning for both the thickness and morphology of the coating. The final COF exhibited a good crystallinity, with stacked 2D-COF sheets, and good conductivity due to the conjugated macrocycle of the porphyrin ligand. The use of EE-SPME resulted in improved selectivity towards PAEs, even in the presence of interfering compounds, including vitamin C, bovine serum albumin, hemoglobin, aromatic amines, PAHs, and hydroxyl-PAHs. The application of a constant positive voltage increased the extraction of negatively charged PAEs while oxidizing hydroxyl-PAHs to aldehydes. The π−π stacking of porphyrin between the 2D adjacent layers produced a high value of charge carrier mobility, thus improving the extraction capability, whereas the amino groups interacted with the analytes by hydrogen bonding. The EE-SPME-GC-MS method was validated for six model PAEs, obtaining LOQs in the 0.2–10 ng/L range and RSDs ≤ 7%. When applied to the analysis of environmental water samples, diisobutyl phthalate, di-n-butyl phthalate, and dihexyl phthalate were detected in lake water in the 45.9−208.6 ng/L range, whereas di-n-butyl phthalate and dihexyl phthalate were detected in the 40.6–178.7 ng/L range in industrial wastewater samples.

Wen and coworkers tested the extraction capability of TpPa-1 for the extraction of synthetic musks from water samples [137]. These compounds are contaminants of emerging concern due to their extensive use, persistence, and bioaccumulation, leading to their widespread presence in environmental and biological matrices. The novel COF coating was synthesized using the solvothermal approach and deposited onto etched SS wires using silicone glue. The material was tested for the DI-SPME-GC-MS/MS analysis of six model synthetic musks, obtaining EFs in the 1214–12,487 range, higher than those achieved using commercial fibers due to hydrophobic interactions and π-π stacking. After validation, LOQs in the 0.04–0.31 ng/L range and RSDs < 10% were obtained. Finally, the DI-SPME-GC-MS/MS was applied for the analysis of tap, underground, and river water, determining contamination levels from 0.72 to 571.1 ng/L. A novel templating strategy based on SiO_2_ nanofibers (SiO_2_ NFs) was developed by Fang and coworkers to synthesize hierarchical COF hollow nanofiber (COF HNFs)-coated stainless-steel fibers for the HS-SPME extraction of both six chlorophenols in water and thymol and carvacrol in milk samples [138]. The very high specific BET surface area (747 m^2^ g^−1^) and the hierarchical porosity of the coating allowed a dramatic improvement in the accessibility of the internal COF micropores, resulting in an overall superior performance of the TpBD-Me2HNFs-12 fiber with EFs in the 452–2632 range and a low matrix effect.

**Table 2:** COF-based coatings used for the SPME extraction of environmental samples.

Analyte	Material	Deposition Method	Extraction Mode	Matrix	Platform	LOD (ng/L)	EFs	References
5 PAHs	BTCH-PTA-COF	sol–gel deposition	HS	beverages and river water	GC-FID	30–50	767–1411	[101]
5 PAHs	TFPA-TAPP-COF	in situ growth	HS	river water	GC-FID	6–24	-	[105]
5 PAHs	porphyrin- COF	physical adhesion	HS	lake water and soil	GC-FID	250–5000	-	[106]
6 PAHs	TAPB-TMC-COF	physical adhesion	HS	river water, pond water, and industrial wastewater	GC-MS	0.29–0.94	819–2420	[107]
7 PAHs	imine- COF-SCU1	in situ deposition	HS	soil	NTD-GC-FID	0.01–0.05 ^a^	-	[94]
6 PAHs	Zn-MOF/COF	physical adhesion	HS	soil	GC-FID	0.1–1c	-	[108]
6 PAHs	Cu-MOF/COF	in situ deposition	HS	soil	GC-FID	0.1–0.5c	-	[109]
6 PAHs and BTEX	2DTP/MIL-101-Cr	physical adhesion	HS	soil	VA-GC-FID	2.1–5 ^a^ (BTEX), 0.07–1.6 ^a^ (PAHs)	-	[110]
7 PAHs	TpBD-COF	in situ electrodeposition	DI	tap water and lake water	GC-FID	1000–5000	-	[111]
8 PAHs	triazine- COF	physical adhesion	IT	tap water, river water, rainwater, and beverages	LC-UV	4–10	1110–2763	[112]
8 PAHs	TpPa-1–1000	physical adhesion	DI	soil	GC-MS	3.1–8.6 ^a^	-	[102]
8 PAHs	g-C_3_N_4_@TpBD	sol–gel deposition	DI	pond water, river water, lake water, well water, rainwater, and snow	GC-MS	20–50	-	[103]
8 PAHs, 4 estrogens and 4 bisphenols	TiO_2_NARs-CFs	in situ growth	IT	tap water, rainwater, and river water	LC-UV	1–10	405–6784	[104]
5 nitroaromatic compounds	MA/PFC-1-HOF	physical adhesion	HS	lake water, river water, and domestic water	GC-MS	4.3–20.8	393–1708	[132]
5 CPhs	TPB-DMTP-COF	physical adhesion	HS	underground, reservoir, and drinking water	GC-MS/MS	0.0048–0.015	1741–4265	[113]
5 CPhs	CuPc-MCOF	physical adhesion	EE-SPME	seawater and seafood	GC-MS/MS	0.8–5	339–988	[114]
2 CPhs, 2-nitrophenol, 2 dimethylphenols	TpBD COF	in situ growth	HS	water and soil	GC-MS	0.39–0.72	11,080–58,762	[115]
6 CPhs	TpBD-Me2HNFs-12	in situ growth	HS	river water	GC-FID	-	452–2632	[138]
BPA	COF-GO	physical adhesion	DI	river water and seawater	CFDI-MS	22.2	-	[97]
5 PBDEs	TpPa-1	physical adhesion	DI	ground water, drinking water, and pond water	GC-NCI-MS	0.0058–0.022	2035–6859	[116]
6 PBDEs	PI@TPB-DMTP	in situ growth	DI	lake water, river water, and wastewater	GC-NCI-MS	0.0083–0.0190	1470–3555	[100]
6 PBDEs	COF-γ-PIL	physical adhesion	DI	lake water, river water, and seawater	GC-MS	0.0021–0.014	913–3625	[117]
6 PBBs	TAPB-DMTP-DB COF	physical adhesion	HS	river water	GC-MS	0.04–0.28	4400–11,360	[118]
TBBPA	TpBD-COF	in situ deposition	DI	tap water, river water, seawater, and beverages	CFDI-MS	0.92	185	[119]
4 TBBPA analoges	TpPaBD_50_-COF	in situ deposition	DI	river water and seawater	CFDI-MS	0.5–12	-	[120]
4 TBBPA analoges	porous-TpBD	in situ deposition	DI	river water and seawater	CFDI-MS	0.1–1	-	[98]
6 PCBs	PAN-SiO_2_@TpPa	physical adhesion	HS	river, lake, and seawater	GC-ECD	0.1–5	2602–5611	[121]
17 PCBs	chlorinated-TpPa-1	in situ growth	HS	seawater, river water, and reservoir water	GC-MS	0.0015–0.0088	699–4281	[122]
15 PCBs	3D TpTAM-COF	physical adhesion	HS	river water and soil	GC-MS	0.001–0.020	5308–10,305	[99]
5 OCPs	Tp-Azo-COF	physical adhesion	DI	tap and well water and beverages	GC-MS/MS	0.002–0.08	1061–3693	[123]
Trifluralin, chlorpyrifos	porous PTA/TAPPT COF	in situ deposition	DI	agriculture wastewater and vegetables	GC-CD-IMS	130, 150	1950, 2123	[124]
Triclosan, methyltriclosn	NiFe_2_O_4_@COF	physical adhesion	DI	tap water, river water, and barreled water	GC-ECD	1–7	279–334	[125]
14 OCPs	COF-CN	physical adhesion	HS	river water	GC-MS/MS	0.0010–13.54	540–5065	[126]
Benzoylurea insecticide	COF-(CF_3_)_2_	physical adhesion	DI	lake water, river water, pond water, wastewater and farmland water	UHPLC-MS/MS	0.06–0.50	44–105	[127]
11 Pyrethroid insecticides	COF_TDBA-TTL_	physical adhesion	DI	river water	GC-MS	0.170–1.68	2584–7199	[128]
8 PFASs	TH-COF	physical adhesion	DI	drinking water, underground water, and river water	UPLC-MS/MS	0.0020–0.0045	-	[129]
14 PFASs	COF-NH-CO-F9	physical adhesion	DI	tap water, river water, lake water, pond water, wastewater, and farmland water	UHPLC-MS/MS	0.0035–0.18	66–160	[130]
14 PFASs	COF-F-1	physical adhesion	DI	lake water and blood	NanoESI-MS	0.02–0.8	105–4538	[131]
5 PAEs	TpTph-COF	in situ growth	DI	lake water and seawater	GC-MS/MS	0.02–0.08	1140–3720	[96]
6 PAEs	porphyrin-based COF	in situ growth	EE-SPME	beverages, lake water, industrial wastewater, and oysters	GC-MS/MS	50–2000	1329 (diethylhexyl phthalate)	[136]
6 Synthetic musks	TpPa-1	physical adhesion	DI	river water, tap water, and underground water	GC-MS/MS	0.04–0.31	1214–12,487	[137]

^a^ ng/g.

## 5. Supramolecular Macrocycles

Supramolecular macrocycles are molecular receptors that can be properly designed to achieve the desired selectivity towards target analytes. Host–guest interactions are based on the lock–key principle [139,140]: the receptors are properly designed to obtain specific interactions and host–guest shape complementarity. In this context, the adsorption of target analytes is obtained by the occurrence of simultaneous non-covalent interactions, such as hydrogen bonding, charge transfer, electrostatic, π-π, van der Waals, and hydrophilic/hydrophobic interactions, and size complementarity between the cavity of the macrocycle and the guest. Among the supramolecular macrocycles, cyclodextrins, calixarenes, and quinoxaline cavitands have been applied as SPME coatings [141] (Table 3).

### 5.1. Cyclodextrins as SPME Coatings

CDs are cyclic oligosaccharides containing D-glucopyranose units bound via α-(1,4)-glycosidic linkages. The shape is an asymmetrical toroid, with primary and secondary hydroxyl groups situated at the top (primary face, narrower) and at the bottom (secondary face, wider) of the system, respectively, resulting in hydrophilic outer faces and a hydrophobic inner cavity. CDs have been applied for the complexation of different compounds, including ions, volatile, and semi-volatile organic compounds [141,142]. These macrocyclic receptors are classified on the basis of the number of oligosaccharide units, with α-CD, β-CD, and γ-CD constituted by six, seven, and eight glucopyranose units, respectively. Considering that α-CDs present a distorted glucose and a smaller cavity and γ-CDs are characterized by higher flexibility and dimensions, resulting in a lower steric barrier, β-CDs are the most commonly used extraction sorbents [141]. CDs can be easily functionalized to improve the complexation capability towards specific classes of compounds, to obtain hybrid nanocomposite materials, or to increase the bonding stability onto the SPME surface. In this context, recent studies have focused on the development of both polymer-based CDs [143] and hybrid nanostructured materials [144,145,146].

**Figure 10:**
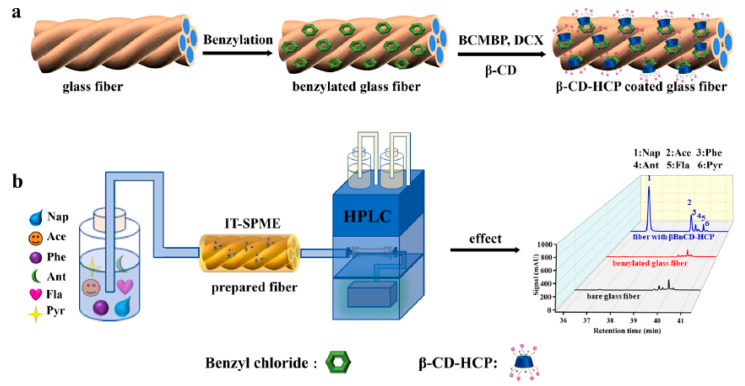
(**a**) Preparation of β-CD-coated glass fiber; (**b**) IT-SPME-LC-UV method for PAHs analysis. Reprinted with permission from [143].

A β-CD-crosslinked polymer was obtained via in situ functionalization of a glass fiber to be used for an IT-SPME extraction [143]. In particular, the glass fibers were benzylated and functionalized using β-CD via the Friedel–Crafts reaction; the coated fibers were then inserted in an SS tube to be used for the on-line IT-SPME-LC-UV analysis of low- and medium-size PAHs (Figure 10). The extraction capabilities of the developed coating were up to 208 times higher than those obtained using an unfunctionalized glass fiber, achieving EFs in the 2130–2670 range. Method validation provided LOQs in the 12–25 ng/L range and a good precision, with RSDs ≤ 7%. Finally, method reliability was tested for the extraction of bottled lake, soil, and tap water spiked with PAHs in the 3–5 μg/L range, obtaining extraction recoveries in the 80–107% range.

In recent studies, multiwalled carbon nanotubes (MWCNTs) were combined with CDs to develop nanostructured hybrid materials [144,145]. The new coatings featured the high selectivity of the supramolecular receptors combined with enhanced surface area/volume ratio and morphological structure of the MWCNTs. In a study by Ghorbani et al., β-CD-functionalized multiwalled carbon nanotubes and acyl chloride functionalized MWCNTs were synthesized and incorporated into a silica composite by the sol–gel technique [144]. A dip-coating process was performed to deposit the hybrid material onto a polypropylene hollow fiber to be used for the SPME extraction of fluoxetine and norfluoxetine, an antidepressant drug and its biologically active form, respectively. The β-CD-MWCNT coating provided a superior extraction capability than that obtained by using only the pristine and acyl-functionalized nanotubes, with EFs of 144 and 151 for fluoxetine and norfluoxetine, respectively. The DI-LC-UV method was validated under optimized conditions, obtaining LOQs of 400 and 300 ng/L and a good precision (RSDs < 10%). The method was applied for the analysis of well, tap, and river water samples, but none of the investigated analytes were detected in the analyzed samples. Similarly, Riboni et al. [145] developed a γ-CD functionalized MWCNT-based material to be used for the extraction of 16 US EPA priority pollutant PAHs from a water sample. In this study, both acidic and hydrogen peroxide pretreatment of the nanotubes were tested, and each of the pretreated MWCNT materials was functionalized using either β- or γ-CDs. Finally, the coatings were obtained by a dip-coating procedure using an epoxy resin. Among the nanocomposite-coated fibers, the highest enrichment capability was achieved by using the H_2_O_2_-treated MWCNTs with γ-CDs as macrocyclic receptors. The achieved results were explained considering both the lower degree of damage of the nanotube surface produced by the hydrogen peroxide, and the steric complementarity between the γ-CD and the analytes, maximizing the host–guest interactions. Under optimal conditions, the DI-SPME-GC-MS method exhibited LOQs in the 0.2–2.3 ng/L range, a linear range of two orders of magnitude, and RSDs ≤ 21%. Exceptionally high EFs, in the 3770(±260)–113,300(±3100) range, were observed. Snow samples collected in the Italian Alpine area were analyzed, detecting a ΣPAHs in the 11.5–34.0 ng/L range.

Finally, Li et al. developed a novel coating using a γ-CD-MOF as an adsorbent material [146]. The MOF was obtained by coordination between γ-CD and potassium ions under alkaline conditions, even though a poor water stability was observed. The fibers were obtained by the dip-coating of SS wires with the pre-synthesized γ-CD-MOF using a neutral silicone sealant; finally, PDMS was deposited onto the surface by means of thermal vapor deposition to obtain a hydrophobic layer. An HS-SPME-GC-MS method was optimized and validated for the extraction of BTEX from aqueous samples. Owing to the high surface area and multifarious nanopores, the developed coating provided an extraction capability up to 11.6 times higher compared to commercial 30 μm PDMS fibers. The method resulted in LOQs in the 0.44–0.95 ng/L range and good precision, with RSDs ≤ 10%. When applied to the analysis of river and pond water, ΣBTEX of 25.6 and 24.6 ng/L were obtained, respectively.

### 5.2. Calixarenes and Cavitands as SPME Coatings

Calixarenes and cavitands are synthetic macrocycles able to host target guests inside their cavity. They are versatile multidentate receptors whose dimensions can be properly modified on the basis of number of monomeric units and whose surface properties can be tuned by the introduction of specific substituents surrounding the hydrophobic cavity. 

**Figure 11:**
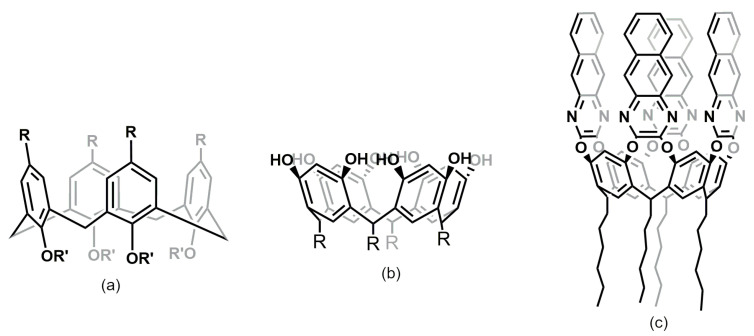
Representation of the structures of (**a**) calix[4]arene, (**b**) resorcinarene-based macrocycle, and (**c**) BenzoQxCav.

Calixarenes are a class of synthetic macrocyclic receptors obtained by the condensation of para-substituted phenol and formaldehyde (Figure 11a). Functionalization can be performed on both faces of the macrocycle, exploiting both the hydroxyl group and the para-substituent of phenols to improve the selectivity of the receptor to anchor the macrocycle onto the substrate or to tune its properties, including the opening and flexibility of the cavity [147]. Najarzadekan et al. developed an SPME coating based on novel polyurethane–polysulfone/calix[4]arene (PU-PSU/calix[4]arene) nanofibers [148] to be used for the HS extraction of 1,2,4-trichlorobenzene, 1,2,3-trichlorobenzene, and 1,2,3,4-tetrachlorobenzene. Four calixarene derivatives, namely, calix[4]arene, sulfonated calix[4]arene, p-tert-butyl- calix[4]arene, and calix[6]arene, were synthesized and electrospun with polyurethane and polysulfone directly onto the SPME fiber. The performance of the different coatings was compared in terms of extraction efficiency, showing the PU-PSU/calix[4]arene had the highest adsorption capability towards chlorobenzenes. After optimization, the HS-SPME-gas chromatography-micro electron capture detector (GC-μECD) method was validated, obtaining LOQs in the 0.4–4.0 ng/L range and RSDs always lower than 10%. Finally, the HS-SPME-GC-μECD method was applied for the quantitation of the target analytes in tap, river, sewage, and industrial water; since no analyte could be detected, the environmental matrices were spiked with the analytes at concentrations of 40 and 400 ng/L, obtaining recoveries in the 80–106% range.

Other important synthetic macrocycles are the resorcinarene-based receptors obtained by the condensation of resorcinol and aldehydes (Figure 11b) [147,149]. As in the case of CDs and calixarenes, the upper rim of the receptors can be functionalized to obtain deeper cavities, tuned polarities, or different complexation capabilities, whereas the substituents at the lower rim are mostly used to tune the macrocycle solubility or to improve the anchoring on a substrate. Very recently, our research group developed and tested a novel cavitand characterized by four benzoquinoxaline walls at the upper rim (BenzoQxCav) as SPME coatings for the extraction of PAHs from water samples (Figure 11c) [150,151]. Compared to the tetraquinoxaline cavitand, the new receptor was characterized by a 2.5 Å deeper hydrophobic cavity, able to host large guests such as PAHs. The coating was obtained by the dip-coating of silica fibers into an epoxy resin followed by dipping into the powdered cavitand. The coating was tested for the extraction of the 16 US EPA priority pollutants PAHs and provided higher enrichment capabilities compared to commercially available PDMS fibers, owing to π-π, CH-π interactions, and analyte engulfment into the hydrophobic cavity. After optimization, the method was validated, obtaining LOQs in the 0.09–1.01 ng/L range, a four order of magnitude linear range, and RSDs ≤ 18%. Finally, the DI-SPME-GC-MS method was applied for the extraction of PAHs from snow samples from Antarctica and the Alps, obtaining ΣPAHs in the 32.2–49.5 ng/L and 10.19–59.13 ng/L ranges, respectively.

**Table 3:** Supramolecular receptor-based coatings used for the SPME extraction of environmental samples.

Analyte	Material	Deposition Method	Extraction Mode	Matrix	Platform	LOD (ng/L)	EFs	References
6 PAHs	β-CD-crosslinked polymer	in situ growth	bottled water, lake water, tap water, and soil water	IT	LC-UV	4–8	2130–2670	[143]
16 PAHs	γ-CD-MWCNTs-H_2_O_2_	physical adhesion	snow	DI	GC-MS	0.1–0.7	3770–113,300	[145]
Fluoxetine and norfluoxetine	β-CD-MWCNTs	physical adhesion	Tap water, river water, and well water	DI	LC-UV	300–400	144–151	[144]
BTEX	γ-CD-MOF	physical adhesion	River water and pond water	HS	LC-MS	0.13–0.29	-	[146]
1,2,4-trichlorobenzene, 1,2,3-trichlorobenzene, 1,2,3,4-tetrachlorobenzene	PU-PSU/Calix[4]arene nanofibers	in situ electrodeposition	Tap water, river water, sewage water, and wastewater	HS	GC-μECD	0.1–1.0	-	[148]
16 PAHs	BenzoQxCav	physical adhesion	snow	DI	GC-MS	0.03–0.30	10,260–125,500	[150]

## 6. Conclusions and Perspectives

Owing to its simplicity, greenness, and extraction capability, SPME is one of the most applied extraction techniques for environmental monitoring. Novel coatings based on supramolecular materials have been developed to increase both the extraction selectivity and enrichment performance, allowing for the quantitation of environmental contaminants at trace and ultra-trace levels. In this context, supramolecular materials have unique features in terms of surface-to-volume ratio, controlled porosity, and tunable surface properties, showing advantages over other materials. A noticeable advantage compared to other nanostructured materials such as alloys, silica-, and carbon-based materials is the possibility of designing a 3D framework with a specific structure and surface chemistry by modifying the linkers, nodes, and monomeric units. Functionalization with phenyl, amino, and ionic groups proved to be particularly suitable for establishing multiple directed interactions between the analytes and the coating, including hydrogen bonding, π-π, electrostatic, and van der Waals interactions. In addition, the porosity of MOF and COF frameworks and the dimensions of the receptor cavity can be tuned at the nm level. Therefore, the extraction is driven by both steric matching and complementarity between the adsorbent and the target molecules, obtaining a unique selectivity and enrichment capability. Future challenges will include the development of alternative approaches that meet the needs of green analytical chemistry, with particular emphasis on solvent-free deposition strategies or coupling with ambient MS techniques for the high-throughput trace determination of environmental contaminants. Another important issue is the pivotal role that machine learning will exert in the synthesis of materials with controlled characteristics. Usually, researchers put a great deal of effort into the development of reliable synthetic strategies, being that this task is laborious and time-consuming. In this context, machine learning can be used as a promising tool for speeding up the development of efficient protocols, reducing the number of experiments, and allowing for a better selection of reagents and experimental conditions.

Finally, the use of density functional theory, simulation, and quality-by-design approaches should be promoted to improve the extraction performance of SPME. 

## Abbreviations

2DTP/MIL-101-Cr	two-dimensional triazine structure/MIL101-Cr hybrid MOF/COF
ALD	atomic layer deposition
APTES	(3-aminopropyl) triethoxysilane
BD	benzidine
BDC	terephthalic acid
BenzoQxCav	tetrabenzoquinoxaline cavitand
BET	Brunauer–Emmett–Teller
BTCH-PTA-COF	hydrazone-based covalent organic framework
BTEX	Benzene, toluene, ethylbenzene and xylenes
CAR	carboxen
CD	cyclodextrin
CD-IMS	corona discharge-ion mobility spectrometry
COF	covalent organic framework
COF-CN	ultrastable crystalline quinoline-linked 2D COF
COF-GO	COF–graphene oxide composite material
COF-PIL	poly(1-vinyl-3-methylimidazolium bis ((trifluoromethyl) sulfonyl) imide)
CPhs	chlorophenols
Cu-DAT	copper-2,5-diaminoterephthalate crystals
CuPc-MCOF	copper-doped phthalocyanine metal covalent organic framework
CVD	chemical vapor deposition
CVR	chemical vapor reaction
DBA	2,5-dimethoxybenzaldehyde
DETA	diethylenetriamine
DI	direct immersion
DMTP	2,5-dimethoxyterephaldehyde
DVB	polydivinylbenzene
EE-SPME	electroenhanced-solid phase microextraction
EF	enrichment factor
g-C_3_N_4_	graphitic carbon nitride
GC-FID	gas chromatography-flame ionization detection
GC-MS	Gas chromatography-mass spectrometry
GC-NCI-MS	gas chromatography-negative chemical ion-mass spectrometry
GC-μECD	gas chromatography-micro electron capture detector
HCNBs	hollow carbon nanobubbles
HOF	hydrogen-bonded organic framework
HPLC-FLD	High-performance liquid chromatography-fluorescence detection
HS	headspace
HZ-PMOF	hollow zirconium-porphyrin-based MOF
IARC	International Agency for Research on Cancer
IL	ionic liquid
IS-VA-HS-SPME	in-syringe vacuum-assisted headspace solid-phase microextraction
IT	in-tube
LC-UV	liquid chromatography-UV detection
LOD	limit of detection
LOQs	limits of quantitation
MAF	metal azolate framework
MC	mechanochemical synthesis
MIL	Matériaux de l′Institut Lavoisier
MIL-101-DETA-F	amino and fluorine dual-functionalized MIL-101(Cr)
MOF	metal organic framework
MS/MS	tandem mass spectrometry
MWCNTs	multiwalled carbon nanotubes
nanoESI-MS	nanoelectrospray ionization-mass spectrometry
NAR	titania nanorod array
N-CNTCs	nitrogen-doped carbon nanotube cages
NH_2_-UiO-66(Zr)-hp	Amino-functionalized UiO-66(Zr)-capped nanocrystals
NH_2_-ZIF-8	amino-functionalized ZIF-8
Ni@NiO/PCs	lotus-like Ni@NiO embedded porous carbons
N_S_ZIF-8^Si^	ZIF-8–superhydrophobic MOF composite material
NTD	needle trap device
OCP	organochlorine pesticide
OPP	organophosphorus pesticide
PA	polyacrylate
Pa	*p*-phenylenediamine
PAE	phthalic acid ester
PAHs	polycyclic aromatic hydrocarbons
PAN	polyacrylonitrile
PAN/Ni-MOF	polyacrylonitrile/nickel-based MOF
PAN/UiO@UiO_2_-N_3_-aptamer	UiO seeded azide aptamer-functionalized MOF
PANI	polyaniline
PBB	polybrominated biphenyl
PBDE	polybrominated diphenyl ether
PCB	polychlorinated biphenyl
PDA	poly(dopamine)
PDMS	polydimethylsiloxane
PES	polyethersulfone
PFASs	poly- and perfluoroalkyl substances
PFOA	perfluorooctanoic acid
PIL	poly(ionic liquid)s
PPCPs	pharmaceutical and personal care products
PPy	polypyrrole
PPy@MIL-101(Cr)	PPy/chromium-based MOF nanocomposite
PU-PSU/calix[4]arene	polyurethane–polysulfone/calix[4]arene
RSD	relative standard deviation
SESI-IMS	secondary electrospray ionization-ion mobility spectrometry
SOM	single-crystal ordered macroporous
SPME	solid-phase microextraction
SS	stainless steel
TAM	(p-aminophenyl)methane
TAPB	1,3,5-tris(4-aminophenyl)benzene
TAPB-TMC-COF	1,3,5-tris(4-aminophenyl)benzene and trimesoyl chloride COF
TBBPA	tetrabromobisphenol A
TFPA-TAPP-COF	tris(4-formyl phenyl)amine-etra(4-aminophenyl)porphyrin COF
TH-COF	dioxin-linked COF
TMC	trimesoyl chloride
Tp	1,3,5-triformylphloroglucinol
Tp−Azo−COF	ketoenamine COF
TpPa-1–1000	1,3,5-triformylphloroglucinol—*p*-phenylenediamine N-doped porous carbons
TpTph-COF	1,3,5-triformylphloroglucinol—4,4′-diamino-p-terphenyl COF
UHPLC-MS/MS	ultra high performance liquid chromatography
US EPA	United States Environmental Protection Agency
ZIF	Zeolitic Imidazolate Frameworks
Zr/N-OMC	zirconium and nitrogen co-doped ordered mesoporous carbon
Zr-MOF@GO	zirconium-based MOF and graphene oxide coating
Σ	total concentration
